# Dynamic metabolic reprogramming in dendritic cells: An early response to influenza infection that is essential for effector function

**DOI:** 10.1371/journal.ppat.1008957

**Published:** 2020-10-26

**Authors:** Svetlana Rezinciuc, Lavanya Bezavada, Azadeh Bahadoran, Susu Duan, Ruoning Wang, Daniel Lopez-Ferrer, David Finkelstein, Maureen A. McGargill, Douglas R. Green, Ljiljana Pasa-Tolic, Heather S. Smallwood

**Affiliations:** 1 Department of Pediatrics, University of Tennessee Health Science Center, Memphis, Tennessee, United States of America; 2 Department of Immunology, St. Jude Children’s Research Hospital, Memphis, Tennessee, United States of America; 3 Center for Childhood Cancer and Blood Disease, The Research Institute at Nationwide Children's Hospital, The Ohio State University School of Medicine, Columbus, Ohio, United States of America; 4 Chromatography and Mass Spectrometry Division, Thermo Fisher Scientific, CA, United States of America; 5 Department of Computational Biology, St. Jude Children’s Research Hospital, Memphis, Tennessee, United States of America; 6 Environmental Molecular Sciences Laboratory, Pacific Northwest National Laboratory, Richland, Washington, United States of America; University of Georgia, UNITED STATES

## Abstract

Infection with the influenza virus triggers an innate immune response that initiates the adaptive response to halt viral replication and spread. However, the metabolic response fueling the molecular mechanisms underlying changes in innate immune cell homeostasis remain undefined. Although influenza increases parasitized cell metabolism, it does not productively replicate in dendritic cells. To dissect these mechanisms, we compared the metabolism of dendritic cells to that of those infected with active and inactive influenza A virus and those treated with toll-like receptor agonists. Using quantitative mass spectrometry, pulse chase substrate utilization assays and metabolic flux measurements, we found global metabolic changes in dendritic cells 17 hours post infection, including significant changes in carbon commitment via glycolysis and glutaminolysis, as well as mitochondrial respiration. Influenza infection of dendritic cells led to a metabolic phenotype distinct from that induced by TLR agonists, with significant resilience in terms of metabolic plasticity. We identified c-Myc as one transcription factor modulating this response. Restriction of c-Myc activity or mitochondrial substrates significantly changed the immune functions of dendritic cells, such as reducing motility and T cell activation. Transcriptome analysis of inflammatory dendritic cells isolated following influenza infection showed similar metabolic reprogramming occurs *in vivo*. Thus, early in the infection process, dendritic cells respond with global metabolic restructuring, that is present in inflammatory lung dendritic cells after infection, and this is important for effector function. These findings suggest metabolic switching in dendritic cells plays a vital role in initiating the immune response to influenza infection.

## Author summary

Dendritic cells are critical in mounting an effective immune response to influenza infection by initiating the immune response to influenza and activating the adaptive response to mediate viral clearance and manifest immune memory for protection against subsequent infections. We found dendritic cells undergo a profound metabolic shift after infection. They alter the concentration and location of hundreds of proteins, including c-Myc, facilitating a shift to a highly glycolytic phenotype that is also flexible in terms of fueling respiration. Nonetheless, we found limiting access to specific metabolic pathways or substrates diminished key immune functions. We previously described an immediate, fixed hypermetabolic state in infected respiratory epithelial cells. Here we present data indicating the metabolic response of dendritic cells is increased yet flexible, distinct from what we previously showed for epithelial cells. Additionally, we demonstrate dendritic cells tailor their metabolic response to the pathogen or TLR stimulus. This metabolic reprogramming occurs rapidly *in vitro* and is sustained in inflammatory dendritic cells *in vivo* for at least 9 days following influenza infection. These studies introduce the possibility of modulating the immune response to viral infection using customized metabolic therapy to enhance or diminish the function of specific cells.

## Introduction

The influenza virus is associated with significant disease burden in the human population and is of particularly high risk to children, the elderly, and those with certain medical conditions, such as pregnancy, obesity, or metabolic disease. In a productive infection, the influenza virus co-opts the host cell’s molecular machinery to induce a virus-centric shift in macromolecular production for budding virions. In connection to this, we previously demonstrated influenza A virus (IAV) infection of human respiratory epithelial cells leads to a fixed hypermetabolic state [1]. Dendritic cells are dispersed throughout the respiratory tract (including the lung parenchyma, alveolar space, and the airway epithelium), are a heterogeneous population with respect to phenotype and function, and are constantly surveying and sampling for pathogens or foreign material at this delicate barrier between the environment and blood stream [2–5]. Following IAV infection, dendritic cells have a critical role linking the innate and adaptive immune responses. These antigen-presenting cells depend on the binding of pathogen-associated molecules to different pattern-recognition receptors, such as toll-like receptors (TLRs), typically engaged on the cell surface or in endosomes. Influenza is sensed intracellularly by TLR3, TLR7, or TLR8, and TLR9, sensing either viral envelope proteins or nucleic acids, respectively [6–12]. These TLRs are expressed at variable levels in human and murine cells of the upper respiratory tract, such as epithelial cells, macrophages, and dendritic cells [13]. The stimulation of these intracellular receptors tailor the antiviral response via retinoic acid-inducible gene-I (RIG-I) and NOD-like receptors or lack thereof, creating a cell-specific response to influenza infection [13]. One molecular driver underpinning immune activation is an increase in metabolism to fuel effector functions requisite for pathogen elimination [14–17]. Recently, seminal papers in the field have demonstrated activation of dendritic cells with the TLR4 agonist lipopolysaccharide (LPS) has profound metabolic consequences that may correlate to altered immune function [17–19]. However, the metabolic response of dendritic cells to influenza infection remains undefined, as does its relationship to TLR stimulation and inactive virus.

To gain a better understanding of the molecular events that initiate the systemic immune response to influenza infection, we performed quantitative proteomics, pulse-chase experiments using labeled metabolic substrates, bioenergetics, and metabolite quantification on influenza A virus strain A/Puerto Rico/8/1934 H1N1 (IAV)-infected and uninfected bone marrow-derived dendritic cells (DC) that were differentiated *in vitro*. Using quantitative proteomics, we found metabolic pathways dominated the enriched protein networks, accompanied by localization changes in metabolic enzymes in response to IAV infection. The network analysis was confirmed with metabolic flux measurements using labeled substrates which showed significant increases in glycolysis and glutaminolyisis. Bioenergetic and metabolite starvation assays demonstrated IAV infected DC increased their metabolic plasticity by reprogramming glycolysis, glutaminolysis, and respiration, better adapting to use diverse fuels to meet the challenges of high energy demand and anabolism needed for activation. We then compared the bioenergetics of IAV infected DC to TLR3, 4, or 7/8 agonists and replication-incompetent IAV, and found IAV infected DC produced significantly more ATP from glycolysis while sustaining non-canonical TCA cycle that was decoupled from glycolysis. Similar to our previous findings in epithelial cells [1], we identified upregulation of the transcription factor c-Myc and found c-Myc inhibition blocked IAV induction of glycolysis and non-canonical TCA cycle. We found inhibiting c-Myc activity or import of specific mitochondrial substrates significantly reduced IAV induced DC motility and T cell activation. We then performed transcriptomics of uninfected versus inflammatory lung dendritic cells 9 days following intranasal IAV infection and found a very significant increase in glycolytic enzymes over those in the TCA cycle. Thus, in response to IAV infection, global metabolic restructuring occurs significantly increasing DC reliance on glucose and glutamine over pyruvate utilization that may have profound functional consequences for the overall immune response to influenza infection depending on the metabolic homeostasis of the lung microenvironment.

## Results

### Influenza induces significant changes in DC protein abundance and localization in key metabolic pathways

**Fig 1:**
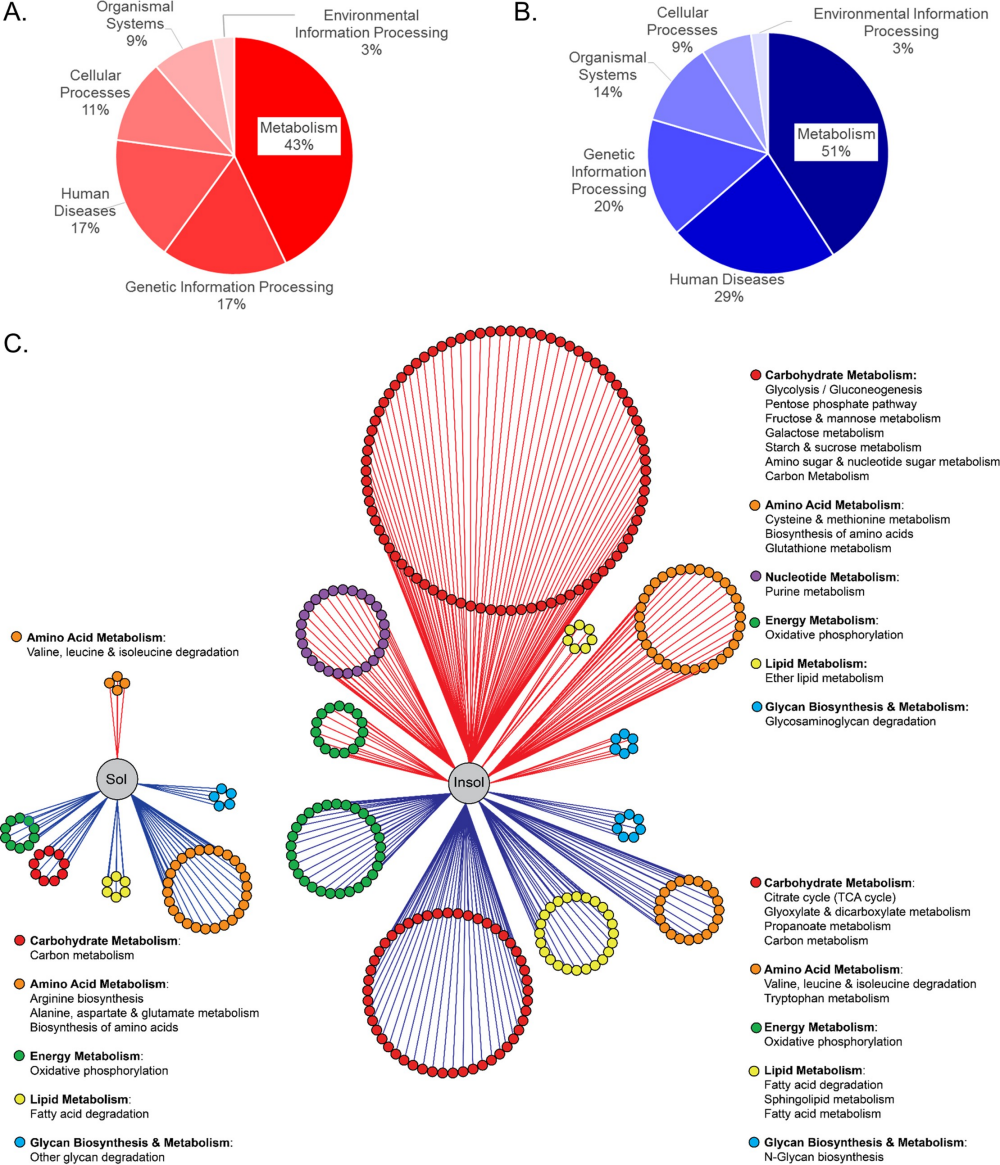
Infection-induced changes in DC protein abundance and localization. Influenza virus strain A/PuertoRico/34 (IAV) was added to DC for 2 hours to allow entry. Then, the medium was replaced, and the infection proceeded for 17 hours followed by cell lysis and protein extraction. Proteins were labeled with either SIL or iTRAQ and subjected to LC-MS/MS. Both proteomes were combined, redundancies removed and confidently identified peptides with abundance changes of 2-fold or greater linked to protein identifiers. The lists of upregulated and downregulated proteins were submitted to the Database for Annotation, Visualization, and Integrated Discovery (DAVID) v6.7 and mapped to KEGG pathways. (A-B**)** Significantly enriched KEGG pathways were grouped by KEGG Orthology (KO) Class for the increased (A) and decreased (B) proteomes. (C) In the network summary of metabolic protein dynamics, the soluble and insoluble proteomes were subdivided by abundance change. Proteins with relative expression changes of two-fold or greater are connected to their location in the soluble or insoluble fraction by red (top) or blue (bottom) edges indicating increased or decreased abundance change, respectively. Each protein node size was held static and color coded by KEGG subclass with the corresponding metabolic pathway of the protein mapped and delineated in the legend. The nodes of each KEGG subclass were arranged into circles that are proportional to the number of proteins with abundance changes in each significantly enriched metabolic pathway.

We used two quantitative labeling strategies to determine proteomic changes after influenza virus infection. We infected DC with a mouse adapted H1N1 IAV strain (A/Puerto Rico/68/34) at a multiplicity of infection (MOI) of 5 for 17 hours. In mouse DC, IAV replication is abortive and has a much lower infectivity rate than that in human DC [20–22]. Thus, we used a higher MOI than in our previous studies and validated effective infection of DC at 17 hours, as determined by viral protein production (S1A Fig). After cells were harvested, soluble and insoluble fractions were subjected to specialized trypsinization strategies to enhance coverage of proteins with complex tertiary structures, [i.e., filter-aided sample preparation (FASP) digestion or several rounds of high intensity focused ultrasound and pressurization(HIFU)] (S1B Fig). The DC proteomes are publicly available in the Proteomics Identifications (PRIDE) repository (PXD013690). Approximately 25,000 peptides were detected with corresponding reporter ions; of these, 7520 mapped to reference sequence accession numbers (Table A S1 File). 70% of the peptides were confidently identified with 2 or more hits and were included in the quantitative analysis. The majority of proteins did not change, 28% responded to infection with ≥ 2-fold change in peptide abundance (S1 Table). Most dynamic changes occurred in the insoluble fraction, with 855 proteins increased and 649 decreased after IAV infection (S1 Table and S1C Fig). To validate the iTRAQ results, we used ^16^O/^18^O labeling and HIFU [23]. This technique identified more than 20,000 peptides, resulting in about 10,000 unique protein accession numbers that mapped to 2,000 proteins. IAV infection induced ≥2-fold change in the abundance of 27% of DC proteins and we found more changes in the insoluble fraction (S1 Table and Tab A S1 File). Although iTRAQ provided expanded coverage of the DC proteome, more than half of all peptides identified by using SIL were also present in the iTRAQ data sets, with similar trends (S1C Fig). Next, we segregated the proteomes into increasing and decreasing sectors (i.e., ≥ 2-fold change), combined the iTRAQ and SIL proteomes, and the most significant peptides were selected. The lists of upregulated and downregulated proteins were submitted to the Database for Annotation, Visualization, and Integrated Discovery (DAVID) v6.8 and queried for enrichment groups [24]. Seventeen hours following IAV infection of DC, we found metabolism accounts for the majority of KEGG Orthology (KO) Class processes and was significantly enriched in both the decreasing and increasing proteomes (Fig 1A & 1B, respectively). Surprisingly, only a small fraction (8.6% up and 11% down) of the enriched pathways were in the immune system or infectious disease KEGG KO subclasses in the BRITE hierarchy (Tab B S1 File).

We identified 432 differentially expressed metabolic proteins that mapped to well-known metabolic pathways (S2 Table and Tab B & C S1 File). Metabolic proteins are also regulated by changes in subcellular location [25–29]. In response to infection, we detected 48 metabolic proteins that moved between the soluble and insoluble fractions (S1 Table). We used a network summary of protein dynamics, as previously described [25], to give a global view of influenza-induced changes in metabolic protein abundance and localization. Proteins were organized based on location in the soluble or insoluble fraction (left and right, respectively) and metabolic proteins were connected to their location by red or blue edges (up or down, respectively) (Fig 1C). Protein coverage per pathway and statistical information for pathway enrichment are provided in Supplementary File 1 Tables B and C. Each protein node was colored by KEGG subclass resulting in a circle size of each KEGG metabolic class that is proportional to the number of proteins with significant fold changes within it (Fig 1C). We found many of the proteins with significant abundance changes were involved in carbohydrate metabolism, which accounted for 47% of the significantly enriched pathways (Fig 1C red nodes and Tab B & C S1 File). In the insoluble increased proteome, we found carbohydrate metabolism had the largest number of proteins dispersed in several significantly enriched KEGG pathways such as pentose phosphate pathway (PPP), fructose/mannose, galactose, starch/sucrose, amino sugar/nucleotide metabolism, and glycolysis (Fig 1C red nodes with red edges). Coverage of glycolytic proteins was complete in both fractions, but we found the majority of soluble proteins increased (S1D Fig green). This was expected, given glycolysis occurs in the cytosol. However, some proteins, including α-enolase, increased in soluble and insoluble fractions due to its role as a glycolytic enzyme and a plasminogen receptor dimerized on the surface of activated monocytes [30]. We validated the increase in α-enolase with microscopy and in both soluble and insoluble fractions with western blots (S1A & S1D Fig). In contrast, carbohydrate metabolism had fewer proteins in the decreased proteomes and these were associated with TCA cycle, glyoxylate/dicarboxylate, and propanoate metabolism (Fig 1C red nodes blue edges). This differential expression may indicate uncoupling of glycolysis from oxidative phosphorylation (OXPHOS). Some proteins appeared to relocate, for example, we found proteins involved in valine, leucine, and isoleucine degradation were decreased in the insoluble proteome but increased in the soluble (Fig 1C orange nodes and Tab B & C S1 File). Conversely, we found key players in the biosynthesis of amino acids decreased in the soluble and increased in the insoluble fractions (Fig 1C orange nodes and Table B & C S1 File). We also found metabolic pathways that were uniformly altered irrespective of location, such as the fatty acid degradation pathway (Fig 1C yellow nodes and Tab C S1 File). Taken together, these results demonstrate DC coordinate changes in metabolic protein abundances and location in response to IAV infection.

### DC restructure metabolic flux after IAV infection

**Fig 2:**
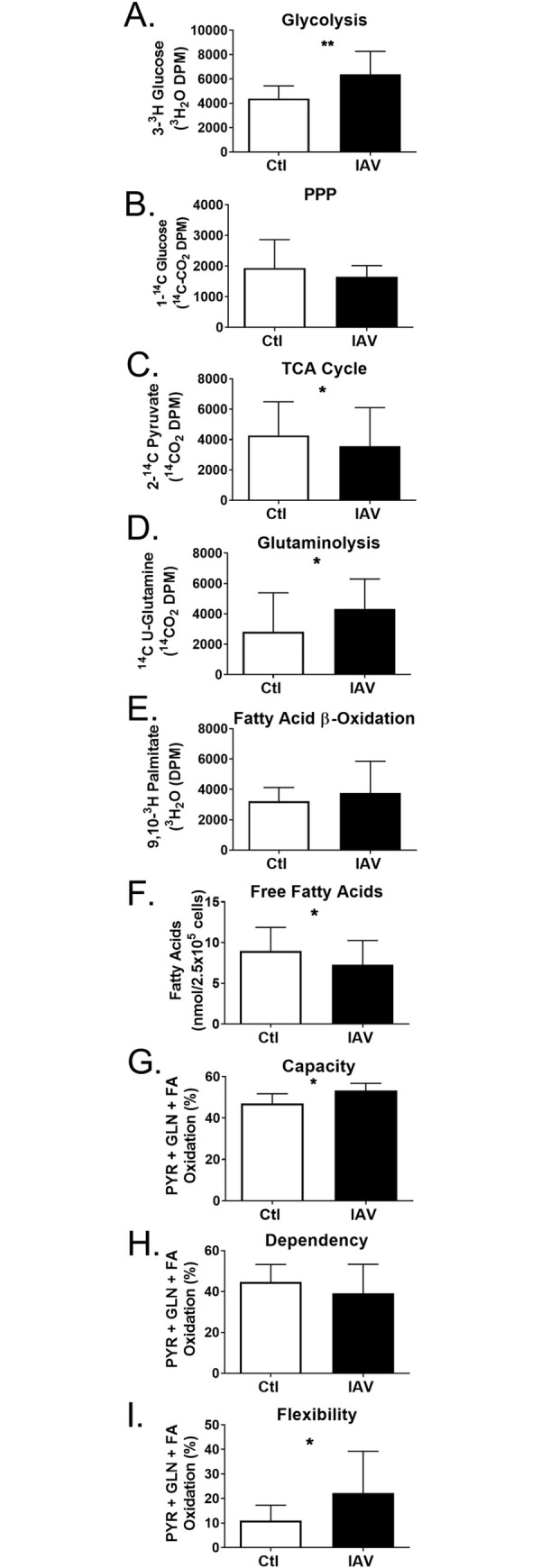
Metabolic changes in infected DC. DC were infected at MOI of 1 for 17 hours. (A-E) Use of isotopically labeled substrates, as indicated on the y axis, were used to monitor the generation of traceable products (^3^H_2_O or ^14^CO_2_) two hours after DC treatments substrates were quenched and allowed to accumulate overnight. Substrates were indicative of the following metabolic pathways: (A) [3-^3^H]glucose (glycolysis), (B) [1-^14^C]glucose (pentose phosphate pathway), (C) [2-^14^C]pyruvate (TCA cycle), (D) [U-^14^C]glutamine (glutaminolysis), and (E) [9,10-^3^H]palmitic acid (fatty acid oxidation). (F) The concentration of fatty acids (≥C8) in DC was determined by a coupled enzyme assay releasing fluorometric product proportional to the fatty acids present and quantifiable with standards after treatments. (G-I) The rates of pyruvate, glutamine, or long chain fatty acids oxidation for respiration were calculated as the percentage of inhibition of oxygen consumption by UK5099, BPTES, or etomoxir, which are specific inhibitors of mitochondrial pyruvate carrier, glutaminase, and carnitine palmitoyltransferase 1A, respectively. Capacity for a specific substrate to drive respiratory OCR was tested by determining baseline OCR, inhibiting the 2 off-target substrates determining OCR, and inhibiting import of the target metabolite. Percent capacity is one minus the baseline OCR less the off-target OCR divided by the baseline OCR less the OCR after all targets inhibited times 100. Dependency on a specific substrate was tested as above reversing the inhibitor sequence and the percent dependence was calculated by deducting the target OCR from the baseline and dividing by the baseline OCR less the OCR after all targets inhibited times 100. Fuel flexibility was calculated as the difference between capacity and dependency. (G) The average capacity of uninfected or infected DC to use pyruvate, glutamine, or long chain fatty acids was determined. (H) The average dependence of uninfected or infected DC on the oxidation of pyruvate, glutamine, or long chain fatty acids was determined. (I) The average flexibility of DC to alternate oxidation of pyruvate, glutamine, or long chain fatty acids was determined for uninfected or infected. The graphs represent the values of two (E-F), three (B-D), or four (A, G-I) independent experiments (3 ≥ technical replicates) and are presented as the experimental mean +/- SD. The normality of these data was tested followed by the appropriate parametric (t-test) or nonparametric (Wilcoxon signed rank test) for normal distributions (A, B, E-H)) or non-normal distributions (C,D,I), respectively. Asterisks correspond to p-values <0.05 (*) and a p-value < 0.01 (**).

To validate the protein network analysis and further characterize influenza-induced changes in metabolism, we quantified glucose, pyruvate, glutamine, and palmitic acid oxidation, as previously described [14]. Isotopically labeled metabolic substrates were used to monitor generation of ^3^H_2_O from glycolysis or beta oxidation and ^14^CO_2_ from the PPP, glutamine utilization, or TCA cycle. Glucose detritiation occurs early in glycolysis when fructose-6-phosphate is converted to fructose-1,6-bisphosphate. By monitoring glucose detritiation, we found influenza infection significantly increased glycolysis (Fig 2A). PPP provides ribose 5-phosphate for nucleotide synthesis and NADPH as a cofactor for fatty acid synthesis. We quantified the oxidative portion of the PPP, following the release of ^14^CO_2_ from glucose-6-phosphate, and found it was unchanged by IAV infection (Fig 2B). Glycolysis is linked to the TCA cycle by the conversion of pyruvate to acetyl-CoA and CO_2_ by the pyruvate dehydrogenase complex. To assess TCA cycle, we quantified the aggregate release of ^14^CO_2_ from [2-^14^C]-pyruvate in 3 steps of the cycle. Consistent with the enrichment of this pathway in the decreased proteome, we found TCA cycle was reduced following influenza infection of DC (Fig 2C). We further examined the pyruvate dehydrogenase complex and found this complex was completely covered in the proteome, and most subunits decreased after infection (S3 Table). Portions of the TCA cycle can oxidize fatty acids or glutamine to produce ATP and metabolic intermediates. Thus, glutamine is a substrate for reductive carboxylation in the non-canonical reversal of the TCA cycle and we found the release of isotopically labeled glutamine increased after IAV infection (Fig 2D). However, fatty acid oxidation (FAO) was unchanged in IAV-infected DC concomitant to a decrease in intracellular free fatty acids (Fig 2E & 2F). Together, the increase in glycolysis and glutaminolyisis with decreased pyruvate utilization in the TCA cycle is consistent with the two major metabolic pathways becoming uncoupled to maintain ATP production while increasing metabolic plasticity [31–36].

Some cancers and T cells display this metabolic profile, which promotes flexibility in mitochondrial substrate oxidation to facilitate macromolecule synthesis while deriving ATP from glycolysis [14, 15, 27, 37–42]. To determine the contribution of pyruvate, palmitic acid, and glutamate to DC bioenergetics, we monitored mitochondrial oxygen consumption rates (OCR) using the XF Mito Fuel Flex Test on an XF Extracellular Flux Analyzer (Xfe96) as previously described [1, 43, 44]. This assay allowed us to determine DC energy utilization, substrate preference, and metabolic plasticity by selectively blocking mitochondrial oxidation of pyruvate, glutamate, or palmitic acid, with UK5099, BPTES, or etomoxir, respectively. High concentration of etomoxir are nonspecific [45], but it was used to target FAO in this assay. The capacity of DC for oxidizing each substrate was determined by quantifying the OCR after blocking the other two substrates, and conversely, dependency on each substrate was determined after inhibiting their individual oxidation (S2A–S2C Fig). Uninfected DC capacity for and dependence on glutamine oxidation was significantly lower than pyruvate or FAO (S2D Fig open bars) but their capacity to use glutamate to fuel mitochondrial respiration was significantly increased after IAV infection (S2D Fig). When we blocked pyruvate entrance into the TCA cycle or FAO, we found a significant reduction in intracellular glutamine in IAV-infected DC, presumably due to increased glutaminolysis (S2E Fig). We then measured α-Ketoglutarate enzyme activity and found it significantly increased after IAV infection when we blocked pyruvate but not FAO (S2F Fig). To determine the net influence of IAV, we combined the percent oxidation capacity or dependence and compared infected and uninfected DC. DC adopted a slight increase in capacity for oxidation of glucose, glutamine, and fatty acids to fuel mitochondrial respiration following infection (Fig 2G) without increasing DC dependence on mitochondrial oxidation of these substrates (Fig 2H). Together, capacity and dependency determine the flexibility of mitochondrial substrate oxidation (i.e., flexibility is the difference between the capacity to use a substrate and the dependency on that substrate). DC increased their flexibility for diverse metabolic substrates after IAV infection (Fig 2I). Collectively, these data indicate IAV-infected DC significantly increase glycolysis without increased shuttling of acetyl CoA from pyruvate into the TCA cycle while also improving their capacity and flexibility for mitochondrial substrate oxidation, including increasing glutaminolysis.

### IAV-infected DC increase glycolytic bioenergetics similar to TLR stimulation

**Fig 3:**
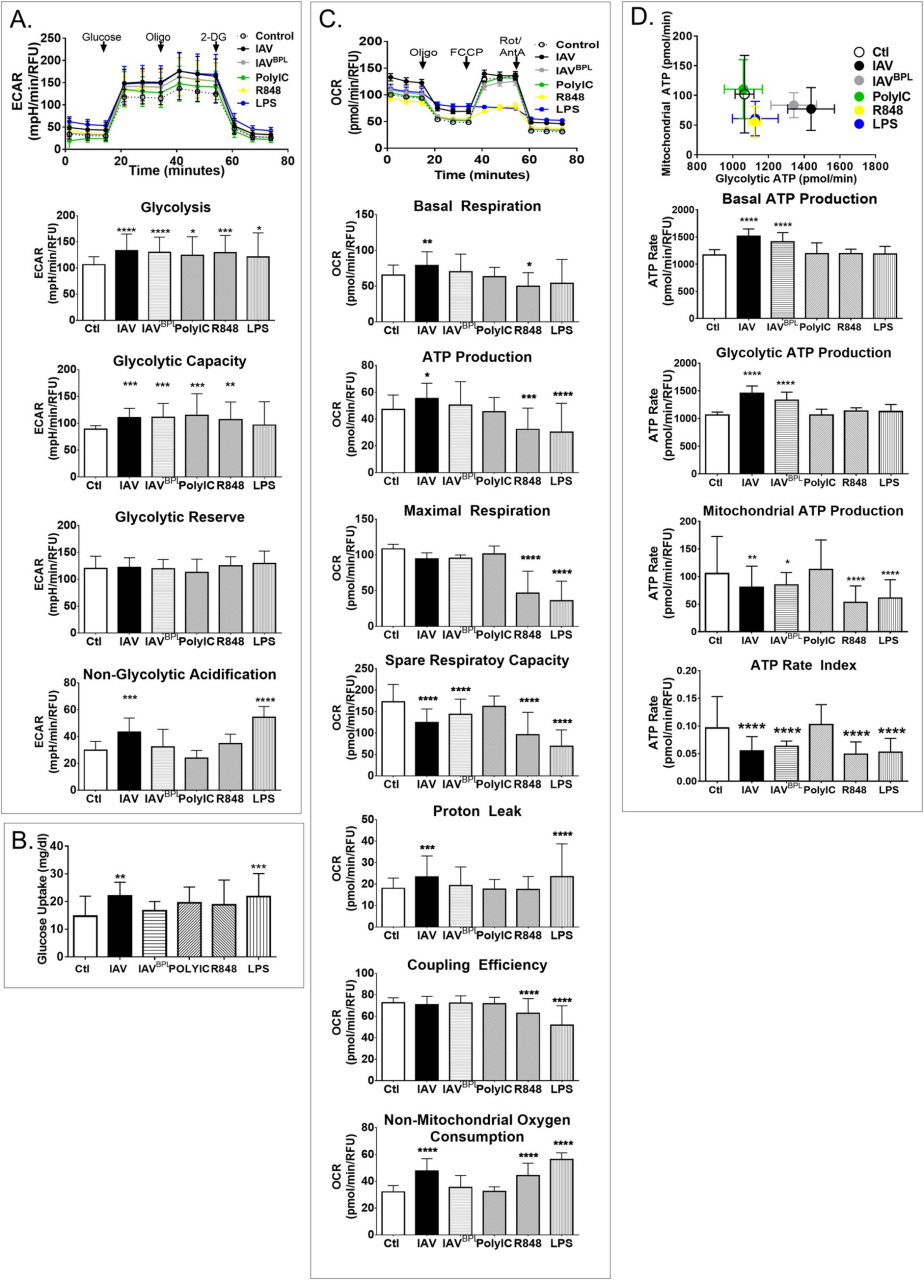
Compared to TLR agonists, IAV infection induces distinct bioenergetics primarily through aerobic glycolysis. (A-D) DC were infected or treated with TLA agonists lipopolysaccharide (LPS), polyinosinic polycytidylic acid (PolyIC), or Resiquimod (R848) for 17 hours followed by metabolic analysis with a Seahorse Xfe96 Flux Analyzer. (A) Glycolytic function was tested with the Glycolysis Stress Test while monitoring real-time extracellular acidification rate (ECAR) with the Xfe96 metabolic analyzer during sequential injections of glucose, oligomycin (Oligo), and 2-Deoxy-D-glucose (2-DG) indicated by arrows. (B) Glucose uptake was monitored from the medium using a standard blood glucometer with glucose standard calibration curves. (C) Mitochondrial respiration was tested with the Mitochondrial Stress Test while monitoring oxygen consumption rates (OCR) in real-time with the Xfe96 metabolic analyzer during sequential injections of oligomycin (Oligo), carbonyl cyanide-p-trifluoromethoxyphenylhydrazone (FCCP), and a mixture of rotenone and antimycin A (Rot/AntA) indicated by arrows. (D) DC maximal mitochondrial ATP changes induced by oligomycin plotted against maximal ATP changes upon glucose depletion determined by respirometry using Xfe96. Each dataset represents the mean of 3 experiments +/- SD for bar graphs or +/- SEM for energetic traces. Significant differences among means were found with ANOVA followed by Tukey and validated with Dunnett’s multiple comparison tests. Tukey test derived p values are symbolized by asterisks indicating adjusted p-values (* p≤0.05, ** p≤0.001, *** p≤0.0001, and **** p<0.0001).

Substrate consumption for ATP production and product efflux vary with cellular metabolism and can be used to measure bioenergetic states. Lactic acid excretion per unit time in glycolysis accounts for the majority of pH change in most cell types, and glycolysis correlates well with extracellular lactic acid accumulation (i.e. R^2^ = 0.9101) [46, 47]. Thus, we used the Xfe96 to determine the extracellular acidification rate (ECAR) as an indirect measure of glycolysis. However, phagocytes employ radical generation and pH in signaling for innate effector functions, so we used the glycolytic stress test to isolate glycolytic ECAR [47–49]. In keeping with our flux analysis, infected DC significantly increased glycolysis, resulting in a mean difference of 26.5242 95% CI [1.62, 51.43] (Fig 3A). Although previous reports indicated detritiation of D-[3-^3^H]-glucose underestimates glycolysis [50], we found IAV-induced increases in glycolytic flux and bioenergetic measures of glycolysis were comparable (Figs 2A and 3A). We also infected DC with the 2009 pandemic H1N1 influenza A/California/04/2009 (CA04) and the H3N2 subtype influenza reassortment virus X31 (i.e. HA and NA genes of A/Hong Kong/1/1968 in the background of PR8). Similar to PR8 (Figs 2A & 3A-IAV), CA04-infected DC significantly increased glycolysis while the increase by X31 was not significant (S4A Fig).

To distinguish cell-mediated responses to viral entry and innate recognition and to determine if DC restructure glycolysis equally with activating stimuli, we used TLR agonists and inactivated IAV. We used imidazoquinoline R848 (R848) to activate both TLR7 and 8 along with MyD88, a compound that is structurally similar to IAV dsRNA [i.e. polyinosinic polycytidylic acid (PolyIC)] to activate TLR3, and lipopolysaccharide (LPS) to activate TLR4, which is activated when DC detect IAV-infected dead cells. Influenza replicates its genome and produces viral proteins within DC, but does not release virions [21, 22]. β-propiolactone (BPL)-inactivated IAV (IAV^BPL^) attaches, fuses, and stimulates TLR7 in the endolysosome without genome replication [51–53]. Like IAV, these treatments significantly increased glycolysis (Fig 3A). ATP synthase inhibition with oligomycin decreases the ATP/ADP ratio, thereby increasing glycolysis to maximal output and allowing us to calculate the glycolytic capacity of DC (i.e., maximal ECAR post oligomycin with less non-glycolytic acidification). The glycolytic capacity was increased by IAV infection, PolyIC, and R848 but not by LPS (Fig 3A). The difference between the maximal glycolytic output and basal glycolysis is the glycolytic reserve, which remained constant across treatments (Fig 3A). The final injection of 2-DG allowed us to quantify any extracellular acidification occurring independent of glycolysis. We found non-glycolytic acidification was significantly increased by active influenza infection and LPS treatment (Fig 3A and S3A Fig). We then quantified glucose uptake to determine if substrate import was coordinated with the increase in glycolysis. IAV and LPS were the only treatments to significantly increased glucose uptake (Fig 3B and S3B Fig). Interestingly, glucose uptake in DC infected with active IAV was significantly higher than inactivated IAV (S3B Fig**)**. Thus, in response to IAV infection DC significantly increase both glucose uptake and glycolysis and the latter was true for all activating stimuli.

### IAV-infected DC mitochondrial respiration is distinct from TLR stimulation

We used Xfe96 respirometry to quantify and isolate the individual components of mitochondrial respiration with the Mitochondrial Stress Test (Agilent) [54, 55]. The Xfe96 quantifies the amount of oxygen spent during respiration, which is stoichiometrically related to the amount of ADP and substrate consumed, and the assay uses sequential inhibition of complex V (ATP synthase) and complexes I and III of the electron transport chain (ETC) and an uncoupler to activate proton conductance allowing one to isolate components of respiration. We found IAV infection significantly increased DC basal respiration, resulting in a mean difference of 22.51 pmol/min/RFU with a 95% CI [3.48, 41.54] between IAV and control (Figs 3C and S3C). Conversely, PolyIC, LPS, and R848 reduced DC basal respiration by 2.3, 7.6, and 12.4 pmol/min/RFU, respectively, and were significantly lower (p ≤ 0.0033) than IAV (Figs 3C and S3C). We found modest but reliable increases in respiration when DC were infected with either CA04 or X31(S4B Fig). Therefore, after IAV infection DC increase basal respiration and this response is significantly different from PolyIC, LPS, or R848 treatments.

After three basal respiratory measures, the ATP synthase inhibitor oligomycin was added to isolate OCR linked to ATP production. IAV infection induced a modest increase in oxygen consumption linked to ATP synthase activity in DC, whereas LPS and R848 treatments significantly reduced it (Fig 3C). These opposing responses resulted in significant differences in respiratory ATP production between IAV-infected and PolyIC-, LPS-, or R848-treated DC (S3C Fig). To maximize respiratory OCR, the protonophore carbonyl cyanide-p-trifluoromethoxyphenylhydrazone (FCCP) is added. This uncouples oxygen consumption from ATP production allowing one to calculate maximal respiration, which was significantly impaired for DC treated with LPS or R848 but unperturbed by IAV infection (Fig 3C). The difference between maximal and basal respiration, the spare respiratory capacity, indicates how closely cells are operating near their OXPHOS threshold. Untreated and PolyIC-treated DC had low basal and high maximal respiration, resulting in similar spare respiratory capacity (Fig 3C). DC treated with either R848 or LPS failed to respond to FCCP resulting in very low maximal respiration and spare respiratory capacity (Fig 3C and S3C Fig). In contrast, IAV-infected DC increased OCR when FCCP activated proton conductance but showed no change in maximal respiration (Fig 3C). The mean difference in IAV-infected DC and untreated control DC spare respiratory capacity was -40.62 pmol/min/RFU with 95% CI [-69.16, 12.09], a significant difference P < 0.0001 (S3C Fig). Therefore, IAV-, LPS-, and R848-treated DC had significantly reduced spare respiratory capacity but different responses to FCCP. Thus, due to higher basal respiration, IAV infected DC were already operating closer to their maximal respiratory output, thereby diminishing their capacity to increase respiration, whereas LPS-stimulated DC had a well-documented respiratory collapse [18, 19] and did not respond to uncoupling (Fig 3C top).

The final inhibitor combination of rotenone and antimycin A blocks proton transport in the ETC for the quantification of proton leak and residual OCR from other sources (e.g., NADPH oxidases, arachidonic acid metabolism, and NOX enzymes). Proton leak is the respiratory OCR that is not coupled to respiratory ATP production. It is unaffected by variations in ATP turnover, unresponsive to fluctuations in substrate oxidation, and can indicate impaired respiration [56]. Active IAV infection and LPS treatment showed significantly more proton leak than controls (Fig 3C). Mitochondrial coupling efficiency is the amount of ATP produced through the ETC per reduction of consumed oxygen. This measure is similar to the traditional phosphate oxygen ratio, and both are sensitive to mitochondrial dysfunction [56]. However, the respiratory coupling efficiency of DC was stable for IAV, IAV^BPL^, and PolyIC, in contrast to LPS and R848 treatments which were significantly reduced (Fig 3C). The impaired FCCP response of both LPS and R848 and diminished coupling efficiency indicate mitochondrial damage, consistent with current reports on the impact of LPS on DC respiration [18, 19].

Activate phagocytes produce reactive oxygen species (ROS) to kill pathogens, but ROS can also damage mitochondria[57]. Enzymes such as cyclooxygenases, lipoxygenases and NADPH oxidases produce ROS, NADPH oxidases alone can account for up to 90% of oxygen consumption by activated phagocytes [58]. ROS may be the source of mitochondria damage, as evidenced by the lack of FCCP response for R848 and LPS (Fig 3C). Indeed, we found non-mitochondrial oxygen consumption was significantly increased by R848, LPS, and active IAV influenza infection (Fig 3C). Impairment of the ETC increases proton leak, lowers coupling efficiency, mitochondrial ATP production, and spare respiratory capacity. Even so cells, including IAV infected DC, can still meet their metabolic demands if basal respiration is elevated and this reduction in dynamic range may limit dramatic ROS bursts [58]. This is probably not the case for R848 and LPS, given they were unresponsive to FCCP and had very significant increases non-mitochondrial OCR.

The mitochondrial stress test indicates IAV-infected DC increase basal oxygen consumption associated with mitochondrial respiration as well as loss of respiratory performance that was also found with R848 and LPS treatments, although the latter demonstrated impaired maximal respiration and coupling efficiency. In lieu of mitochondrial impairment, these measures of pyruvate flux, glutamine flux, and respirometry showed influenza infection significantly reduced normal pyruvate oxidation while increasing glutamine oxidation for fueling the TCA cycle. This finding is reasonable considering that 17 hours after IAV infection, DC had significantly depleted glucose in their surroundings (i.e., a mean difference of -6.680 mg/dl glucose with 95% CI [-12.24, -1.115]) (Fig 3B). Furthermore, DC are known to shift to glutaminolysis to fuel TCA cycle to support OXPHOS when glucose-derived pyruvate is lacking [19, 58–62] which we found was enhanced by IAV infection (S2D and S2E Fig). Thus, even though IAV infection significantly increased DC glycolysis, it also reduced pyruvate dehydrogenase, concomitant to a modest increase in glutaminolysis acting to fuel OXPHOS-related oxygen consumption without evidence of mitochondrial damage (FCCP response or coupling efficiency).

### IAV-infected DC increase ATP production via glycolysis distinct from TLR agonists

To determine the source of ATP production in DC, we measured total ATP production rates from glycolysis or mitochondrial OXPHOS using the Seahorse XFp Real-Time ATP Rate Assay (Agilent). Changes in the relative output of ATP from glycolysis and mitochondrial respiration often reflects the cell’s metabolic phenotype and can be derived from the ECAR and OCR after the addition of oligomycin and a mix of rotenone and antimycin A. The rate of ATP production from mitochondrial ATP synthesis can be quantified after oligomycin treatment. When mitochondrial respiration is completely blocked, the total proton efflux rate (PER) can be used to calculate the glycolytic ATP production rate. Consistent with the above bioenergetic measures, DC fell into three distinct metabolic phenotypes: hyper metabolic IAV and IAV^BPL^, metabolically impaired R848 and LPS, and normal uninfected control and PolyIC (Fig 3D top). DC significantly increased total ATP production in response to IAV infection, irrespective of BPL inactivation (Fig 3D). Following IAV infection, DC had a striking 39% increase in glycolytic ATP production, resulting in a mean difference of 418.6 pmol/min/RFU with 95% CI [297.1, 540.1] (Fig 3D & S3D Fig). In contrast, R848 or LPS treatment significantly reduced mitochondrial ATP (Fig 3D & S3D Fig). One hallmark of a fundamental shift in metabolic phenotype is a change in the ATP ratio from mitochondrial to glycolytic ATP production. The ATP rate index significantly decreased in DC following IAV infection (Fig 3D bottom). This was due to the very significant increase in glycolytic ATP production concomitant to modest changes in mitochondrial ATP, indicating DC change to a more glycolytic and less oxidative phenotype following IAV infection. In contrast, DC activated with LPS or R848 also exhibit a dramatic decrease in the ATP rate index, but this was due to a significant reduction in mitochondrial ATP production without a change in glycolytic ATP (Fig 3D). Taken together, these results indicate activated DC re-wire metabolic pathways differently depending on IAV infection or TLR stimulation.

### Regulation of IAV induced DC metabolic phenotype

**Fig 4:**
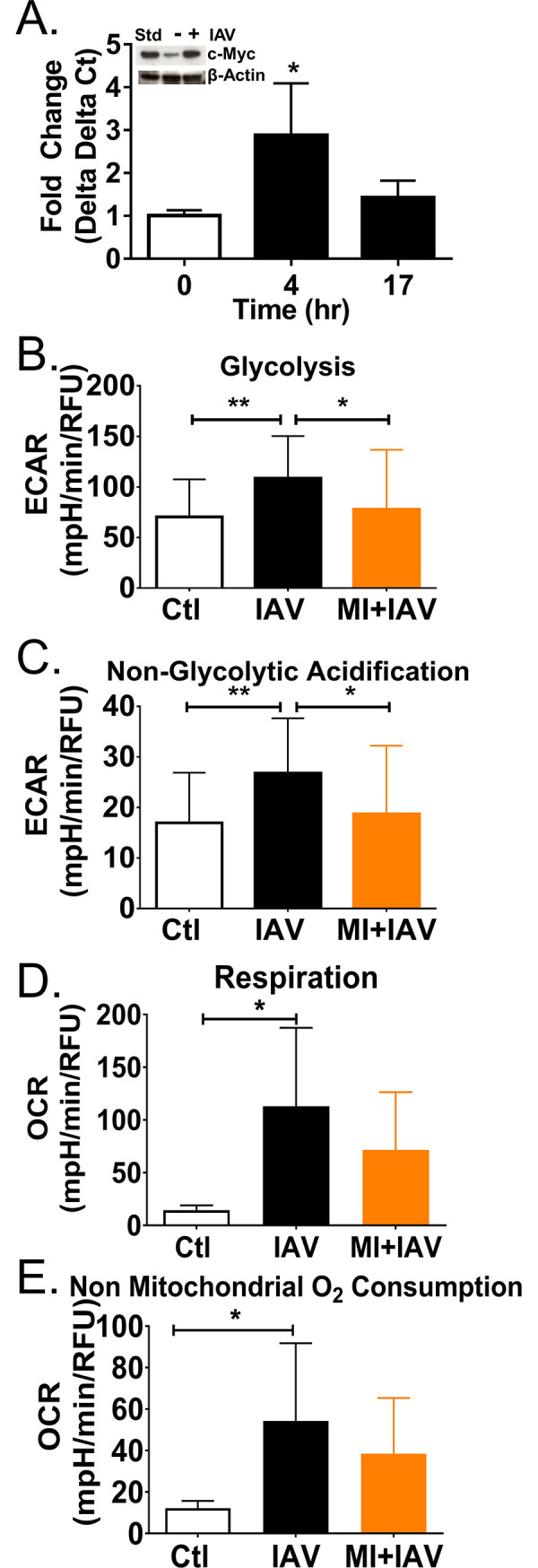
c-Myc inhibition blocks IAV-induced glycolysis. (A) Up to 17 hours PI, qPCR and immunoblotting of RNA and lysates were performed and quantification of target gene expression normalized to β-actin using the 2−ΔΔCt method. One representative blot is presented in the inset. (B-E) DC were seeded in Seahorse XFe-96 plates and pretreated with 2 μM cMyc inhibitor (MI) for 4 hours then IAV infected for 17 hours. (B-C) Glycolytic function was tested with the Glycolysis Stress Test while monitoring real-time extracellular acidification rate (ECAR) with the Xfe96 metabolic analyzer during sequential injections of glucose, oligomycin (Oligo), and 2-Deoxy-D-glucose (2-DG). Data represent means ± SD of 4 experiments. (D-E) Mitochondrial respiration was tested with the Mitochondrial Stress Test while monitoring oxygen consumption rates (OCRs) in real-time with the Xfe96 metabolic analyzer during sequential injections of oligomycin (Oligo), carbonyl cyanide-p-trifluoromethoxyphenylhydrazone (FCCP), and a mixture of rotenone and antimycin A (Rot/AntA). These graphs represent the mean values of 3–4 independent experiments +/- SD. Significant differences among means were found with ANOVA followed by Tukey's multiple comparisons test with asterisks indicating adjusted p-values (* p≤0.05 and ** p≤0.001).

One transcription factor that promotes glycolysis and glutaminolysis in cancer and activated T lymphocytes is c-Myc [14, 63–66]. DC express variable levels of three Myc family members (c-Myc, n-Myc, and l-Myc) that regulate immune cell development, differentiation, and activation [14, 67, 68]. We found IAV infection induced transient c-Myc expression peaking at 4 hours (Fig 4A). c-Myc protein levels also increased 17 hours post infection compared to uninfected controls (Fig 4A inset). We probed for hexokinase, the enzyme involved in the rate limiting step of glycolysis, which increased similar to c-Myc while we found no change in l-Myc (DNS). Many c-Myc driven cancers are sensitive to glucose and glutamine deprivation resulting in rapid cell death; this is referred to as glucose and glutamine addiction [63, 65, 66, 69–71]. We previously found c-Myc transiently increased in IAV-infected epithelial cells and they were glucose and glutamine addicted [1]. In the absence of glucose, the proportion of viable DC dropped (S4C Fig open bars). However, neither infection nor activation with TLR agonists increased cell death (i.e., reduce the live to dead ratio) in the absence of glucose (S4C Fig). The same was true when glutamine was restricted, except for R848 and LPS which increased the live-to-dead ratio (S4C Fig). Removal of both glutamine and glucose had no adverse effect on IAV-infected DC (S4C Fig open and solid bars). LPS was the only treatment to slightly increase DC death with glutamine and glucose deprivation (S4C Fig red vertical stripes). We observed high levels of viability with very little cell death in all treatments, indicating that metabolic differences detailed above were not due to population decline or adverse effects from treatment with high concentrations of agonists. Furthermore, the viability of infected DC was more stable with varying metabolite levels than we previously observed in IAV-infected respiratory epithelial cells [1]. These findings were unexpected, given the transient rise in c-Myc (Fig 4A), but were consistent with DC not developing specific substrate dependency while acquiring increased metabolic flexibility following infection (Fig 2G & 2I).

To determine if DC require c-Myc activity to modulate their metabolic response to infection, we treated DC with c-Myc inhibitor (MI), also known as 10058-F4, for 4 hours and infected them. c-Myc inhibition significantly reduced the IAV induced increase in glycolysis, similar to uninfected levels (Fig 4B) as well as non-glycolytic acidification (Fig 4C). Increases in c-Myc primarily boost metabolism through the glycolytic pathway by increasing levels of lactate dehydrogenase A, glucose transporter 1, phosphoglucose isomerase, phosphofructokinase, glyceraldehyde-3-phosphate dehydrogenase, phosphoglycerate kinase, and enolase [72–74]. We found many of these proteins increased in the proteome (S1A & S1D Fig). However, more recent studies have indicated c-Myc also regulates mitochondrial metabolism and biogenesis [66, 75–77]. We quantified basal respiration and non-mitochondrial oxygen consumption and found MI treatment had a modest impact after infection (Fig 4D and 4E). Thus, DC increased c-Myc expression and glycolysis in response to IAV infection and inhibiting c-Myc activity blocked the IAV-induced glycolytic increase.

### Metabolic rewiring in IAV infection is necessary for DC effector functions

**Fig 5:**
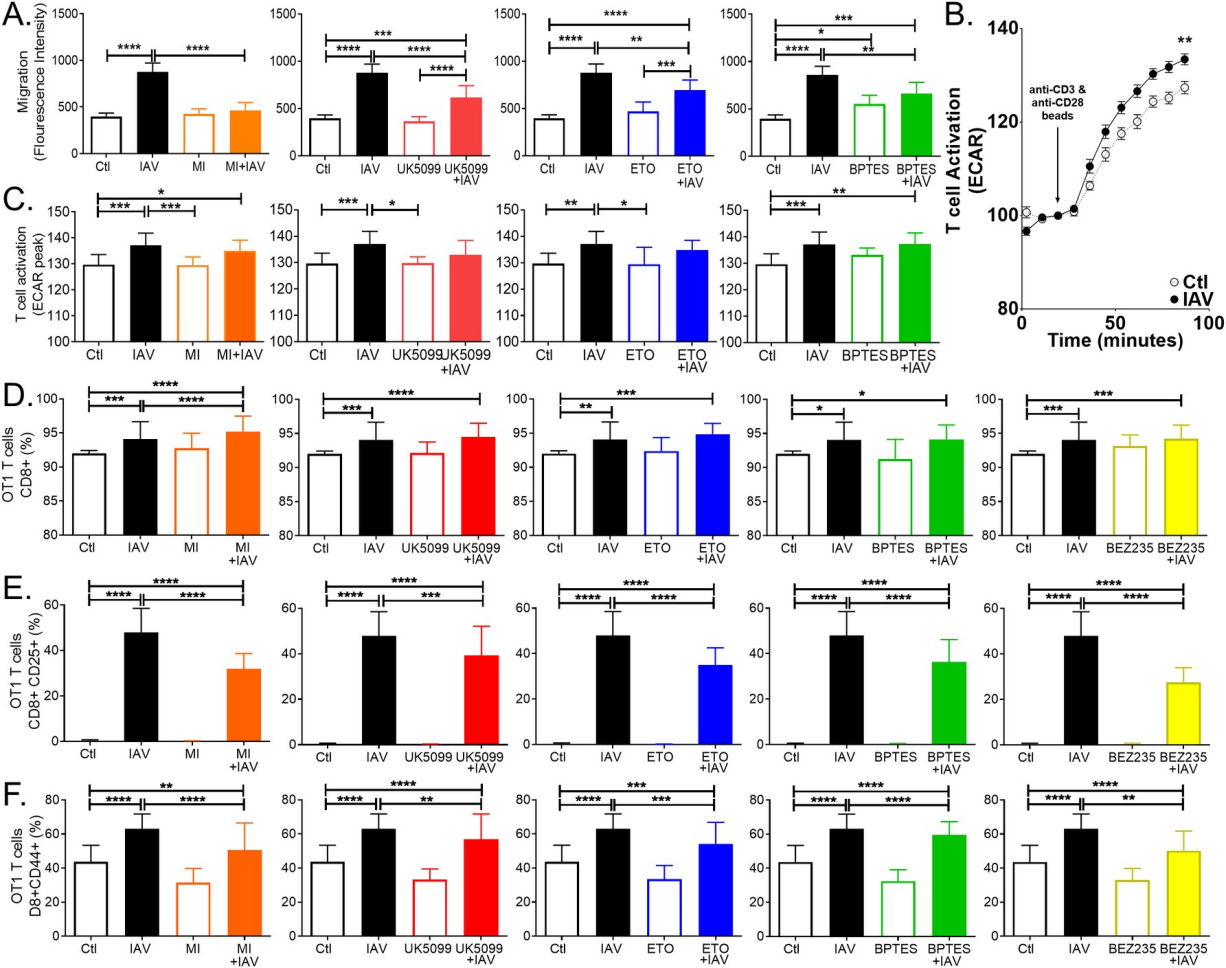
Limiting glycolysis or mitochondrial oxidation of pyruvate, glutamine or long chain fatty acids impairs infected DC function. (A) DC were seeded on precoated Oris 96-well motility plate and allowed to adhere for 18+ hours followed by 4 hours of inhibitor treatment (2 μM c-Myc inhibitor, 3 μM BPTES, 4 μM etomoxir or 2 μM UK5099), plug removal and infection (MOI = 5) for 17 hours. Cells were stained with Calcein-AM (2 μM), and viability and motility were determined with fluorescence at 485/528 nm. The graph represents the mean values of 3 experiments +/- SD. (B-C) CD8+ T cells were isolated by negative depletion from fresh splenocytes of female Ot1 mice and co-cultured with DC at 5:1 ratio T cells to DC for 24 hours. Basal ECAR was established, and T cell activation by anti-CD3/CD28-coated DynaBeads was monitored for 2 hours and maximal ECAR determined. (B) One representative graph is shown from 3 independent experiments. (C) The graph represents the mean values of 3 experiments +/- SD. (D-F) CD8+ T cells were isolated by negative depletion from fresh splenocytes derived from C57BL/6-Tg(TcraTcrb)1100Mjb/J (OT1) and co-cultured with DC at 5:1 ratio of T cells to DC for 24 hours. T cell were then stained for CD8, CD45.1, CD25, and CD44 and enumerated with flow cytometry. We selected CD8 and CD45.1 positive (D) and then gated on CD25 (E) and CD44 (F). The graph represent the mean values of 3 experiments +/- SD. Significant differences among means were found with ANOVA followed by Tukey's multiple comparisons test with asterisks indicating adjusted p-values (* p≤0.05, ** p≤0.001, *** p≤0.0001, and **** p<0.0001).

DC migration from the lungs to the lymph nodes is a critical first step in bridging the innate and adaptive responses to IAV and for inducing the IAV virus-specific CD8 T cell response requisite for viral elimination [5]. Increases in DC trafficking occur quickly after IAV infection and peak *in vivo* around 18 hours [78, 79]. We used a fluorescent cell motility assay to quantify DC motility in response to IAV infection (S5A Fig). DC significantly increased motility after IAV infection (Fig 5A black). Myc inhibition had no effect on uninfected DC motility but completely blocked IAV-infected DC motility (Fig 5A orange)**. I**nhibiting mitochondrial oxidation of pyruvate or FAO had no impact on uninfected DC motility and significantly reduced infected DC motility, but it remained significantly higher than uninfected DC (Fig 5A red and blue). This was also true for inhibiting glutamine oxidation, but we found this also slightly increased uninfected DC motility (Fig 5A green). Although the role of DC phagocytosis post infection is unclear and likely minimal, we then sought to determine if phagocytic activity of infected DC could be modulated by targeting these same pathways. We found no differences among uninfected drug-treated controls and found no effect after IAV infection (S5B Fig). Thus, inhibiting c-Myc ablates IAV-induced DC motility, while blocking mitochondrial oxidation of pyruvate, glutamine, or fatty acids significantly impairs it.

Next, we used flow cytometry to characterize the impact of these metabolic inhibitors on DC populations following IAV infection. We selected CD11c and MHCII positive DC and then gated on CD40, CD80 and CD86. We found inhibition either pyruvate oxidation or FAO significantly reduced MHCII positive IAV infected DC (S5C). In contrast, there was no change in CD40 (S5D Fig). IAV and IAV infected DC treated with UK5099 significantly increased CD80 positive DC levels, the rest of the metabolics inhibitors blocked surface expression enough to be indistinguishable from controls but not enough to be significantly different from IAV infection alone (S5E Fig). In contrast, only IAV infected DC had significantly higher levels of CD86 positive DC, and all IAV infected DC treated with metabolic inhibitors had significantly lower CD86 positive DC (S5F Fig). In keeping with our motility study, c-Myc or FAO inhibition significantly reduced the levels of CCR7 positive DC (S5G Fig).

Initial studies of DC function *in vitro* revealed IAV can infect and stimulate DC maturation into potent antigen presenting cells that elicit a robust IAV-specific T cell response relative to other APCs without the addition of exogenous cytokines [80–82]. We used Agilent’s Seahorse T Cell Activation Assay to quantify T cell activation, as previously described [83–86]. This assay uses anti-CD3/CD28 bead stimulation to activate primary splenic T cells [14, 83–87]. We found co-culturing with IAV-infected DC prior to bead stimulation significantly increased their response to anti-CD3/CD28 bead stimulation (Fig 5B). To delineate the roles of c-Myc, glycolysis, and mitochondrial substrate oxidation in DC maturation, we inhibited each before infecting DC with IAV for 17 hours and then co-cultured the DCs with freshly purified wild-type T cells from naïve or homologously primed C57BL/6 mice. Co-culture of T cells with IAV infected DC significantly enhanced the T cell response to bead stimulation irrespective of DC glycolysis or glutaminolysis (Fig 5C orange and green). In contrast, when either pyruvate or FAO was blocked IAV infected DC did not augment the T cells response to stimulation, resulting in activation peaks that were similar to uninfected controls (Fig 5C red and blue).

Following DC-T cell co-culture, we assessed CD8+ T cell populations staining positive for CD8, CD25 and CD44 with flow cytometry (S6A Fig). We performed a time course and found the pretreatment of DC with the inhibitors remained effective after 24 hours of co-culture with T cells but were diminished by 48 hours. Only etomoxir treatment reduced CD8+ T cells (S6B and S6C Fig). DC that were pretreated with metabolic inhibitors reduced the CD25+ T cell population following co-culture (S6B and S6C Fig). However, due to the response heterogeneity of the T cell population, we moved to a transgenic system. Using this system, Sckisel and co-workers found antigen independent proliferating T cells did not up-regulate CD25 and that >90% of antigen specific CD8’s were both CD44+ and CD25+[88]. We purified CD8+ T cells from spleens of C57BL/6-Tg (TcraTcrb)1100Mjb/J (OT1) mice that contain transgenic inserts for Tcra-V2 and Tcrb-V5 genes. OT1 T cell receptor interacts with the ovalbumin amino acid sequence SIINFEKL, corresponding to residues 257–264, in the context of H2Kb. We infected with a PR8 influenza virus reverse engineered to express the SIINFEKL OVA peptide in the neuraminidase stalk (IAV^OVA^), that was generously provided by Dr. Richard Webby [89]. Dendritic cells were treated with vehicle or inhibitor and infected for 17 hours with IAV^OVA^. We used three controls: T cells without DC (-DC), T cells co-cultured with uninfected and untreated control DC (Ctl), and T cells co-cultured with untreated DC infected with IAV^OVA^. We found co-culture of DC infected with IAV^OVA^ significantly increased the population of CD8+ T cells and CD25+ and CD44+ populations of CD8 T cells irrespective of drug treatment. (Fig 5D and S6D Fig). However, consistent with our previous results with wild type mice, we found restricting DC metabolism can significantly reduce the population of CD8+ T cells expressing the early activation marker CD25 as well as CD44 compared to IAV^OVA^ alone (Figs 5E, 5F and S6D). Similar to the T cell activation assay (Fig 5C), we found c-Myc inhibitor and etomoxir had the most profound effects (Figs 5E, 5F and S6D). Based on our previous studies in IAV-infected epithelial cells and DC, we knew the PI3K/mTOR inhibitor BEZ235 would block the IAV-induced increase in glycolysis (1). Thus, we wanted to compare T cell responses to DC treated with this inhibitor. We found BEZ235 significantly reduced CD25- or CD8-positive T cells similar to c-Myc inhibitor (Fig 5E and 5F yellow and orange, respectively). Collectively, these data show specific metabolic pathways are essential to DC functions and glycolysis is critical for IAV induced DC motility and T cell activation.

### Lung TipDC globally reprogram metabolism in response to IAV infection *in vivo*

**Fig 6:**
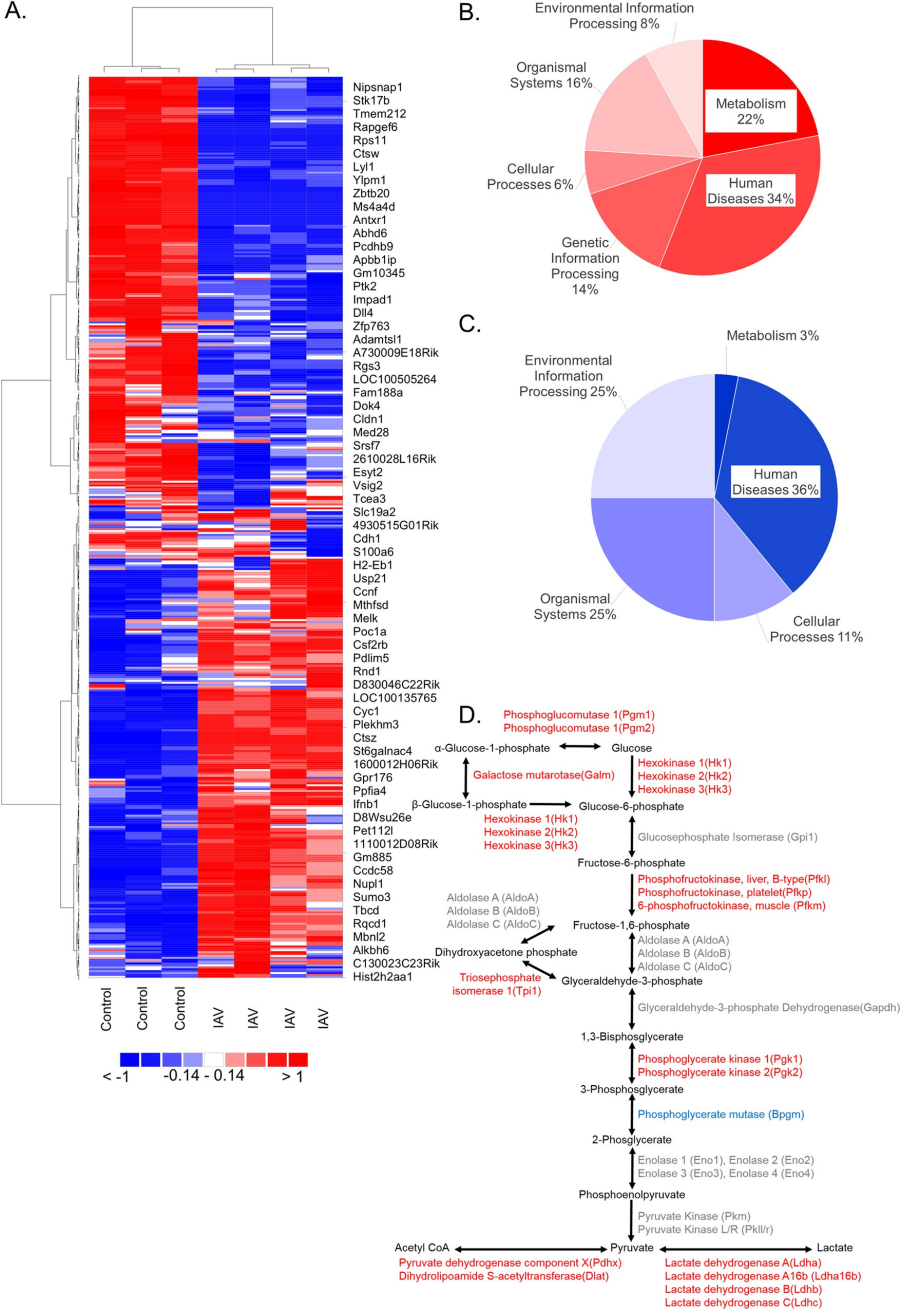
TipDC reprogram metabolism after accumulating in the lungs of IAV-infected mice. At zero or nine days following intranasal infection of mice with IAV (PR8), lungs were homogenized, and cells extracted. Cells were antibody stained for TipDC surface markers CD11b, Ly6c, GR-1, and MHCII. TipDC controls (Ctl) from day 0 were sorted based on high levels of CD11b, Ly6c, and GR-1. TipDC from day 9 of the IAV infection (IAV) were sorted based on high levels of CD11b, Ly6c, GR-1, and MHCII. cDNA libraries were generated from RNA and sequenced. (A) Confidentially identified transcripts were k-means clustered followed by ascendant hierarchical clustering, and genes with standard deviation threshold < 50% were removed. The data values were replaced by corresponding color intensities of blue to red through white based on interquartile range. (B-D) Significant gene expression differences were determined using Tukey’s honest significant difference test for multiple comparisons with a Benjamini-Hochberg post-hoc false discovery rate correction. TipDC genes that were either upregulated (red) or downregulated (blue) following IAV infection were mapped to KEGG pathways and significantly enriched pathways identified and sorted by KEGG Class (B-C). All isoforms of the central enzymes in glycolysis from the TipDC transcriptome are presented in color, including those that were unchanged (gray), increased (red) and decreased (blue).

We speculated that glycolysis and specific mitochondrial substrates that fuel OXPHOS are critical for DC motility and T cell priming *in situ* based on our *in vitro* models and asserted this is likely to affect the immune response during influenza infection [78, 79, 90]. To determine if these metabolic pathways were relevant *in vivo*, we intranasally infected C57BL/6 mice with IAV or mock for 9 days and isolated TNF-α and inducible nitric oxide synthase producing DCs (TipDC) by sorting on CD11b^hi^, Ly6c/GR-1^hi^, MHCII^hi^ surface markers. TipDC are an inflammatory subtype that drive Th1 response and, in the context of IAV infected mice, they present antigen to CD8 T cells and are required for influenza-specific CD8+ T cell proliferation in the lung to mediate viral clearance [91–95]. We purified RNA from TipDC and confidently identified 13,285 transcripts (Tab A S2 File). To evaluate the group trends, sample uniformity and identify potential outliers we employed unsupervised multivariate principal component analysis (PCA). S7A and S7B Fig show the separation of groups by PCA and the selection criterion depicted on a double filtered volcano plot with the statistical effects on the y-axis and biological effects on the x-axis. These data were independently k-means clustered followed by ascendant hierarchical clustering based on Euclidian distances and rearranged with similarity proportional to a closer spatial relationship for DC columns and transcript rows. This approach resulted in clear separation of infected and uninfected TipDC and highlighted the difference in gene expression (Fig 6A). We found 4,788 transcripts were significantly different after IAV infection (Tab B S2 File). We identified fifty pathways that were significantly enriched from these transcripts, including many governing the immune response (e.g., TLR signaling, phagosome, TNF signaling, and phagocytosis), Influenza A, as well as Global Metabolic Pathways (mmu01100), which was the second most significantly enriched (Tab C S2 File). Similar to the *in vitro* differentiated DC proteome (Fig 1A), we found pathways within the Metabolism KEGG Class comprise a large proportion of the increased TipDC transcriptome (Fig 6B). We input the genes from the 11 significantly enriched metabolic pathways into DAVID to identify the overlap of these metabolic proteins and found glycolysis, OXPHOS, purine and amino acid metabolism were some of the pathways these proteins regulate (Tab D S2 File). Fig 6D depicts the enzymes and products of glycolysis with the transcripts that were significantly altered by IAV infection, the majority of these key enzymes were upregulated (red). Thus, among TipDC transcripts that significantly increased after IAV infection, we found eleven metabolic pathways and glycolytic enzymes were significantly enriched (p < 0.00001).

In contrast, metabolism comprised only 3% of the enriched KEGG Classes from TipDC transcripts that decreased following IAV infection (Fig 6C and Tab E S2 File). They were purine metabolism and metabolism of xenobiotics by cytochrome P450. Many pathways enriched in the downregulated transcriptome reflect pathways one would anticipate being suppressed in TipDC on day 9 of the infection [e.g., leukocyte transendothelial motility, extra cellular matrix (ECM)-receptor interactions, cell adhesion molecules (CAMs), focal adhesions]. We were surprised to see the Influenza A pathway enriched in the transcripts that decreased with IAV. On closer analysis, we found this pathway was differentially regulated, as were Cytokine-cytokine receptor interaction and Fc gamma R-mediated phagocytosis (Tab F S2 File). These pathways include increased expression of viral sensors TLR4, TLR3, IFNAR1 and IFNAR2 and related signaling molecules such as MyD88, STAT1, and STAT2 concomitant to decreased expression of FAS, FASLG and TRAIL along with NS1-regulated proteins NFX1, NFX 2, NFX 3, NFX5, PIK3CA, PIK3CB, PIK3CD, PIK3R1, PIK3R2, PIK3R, and PIK3R3. Phosphoglycerate mutase was the only glycolytic transcripts that significantly decreased (Fig 6D blue).

**Fig 7:**
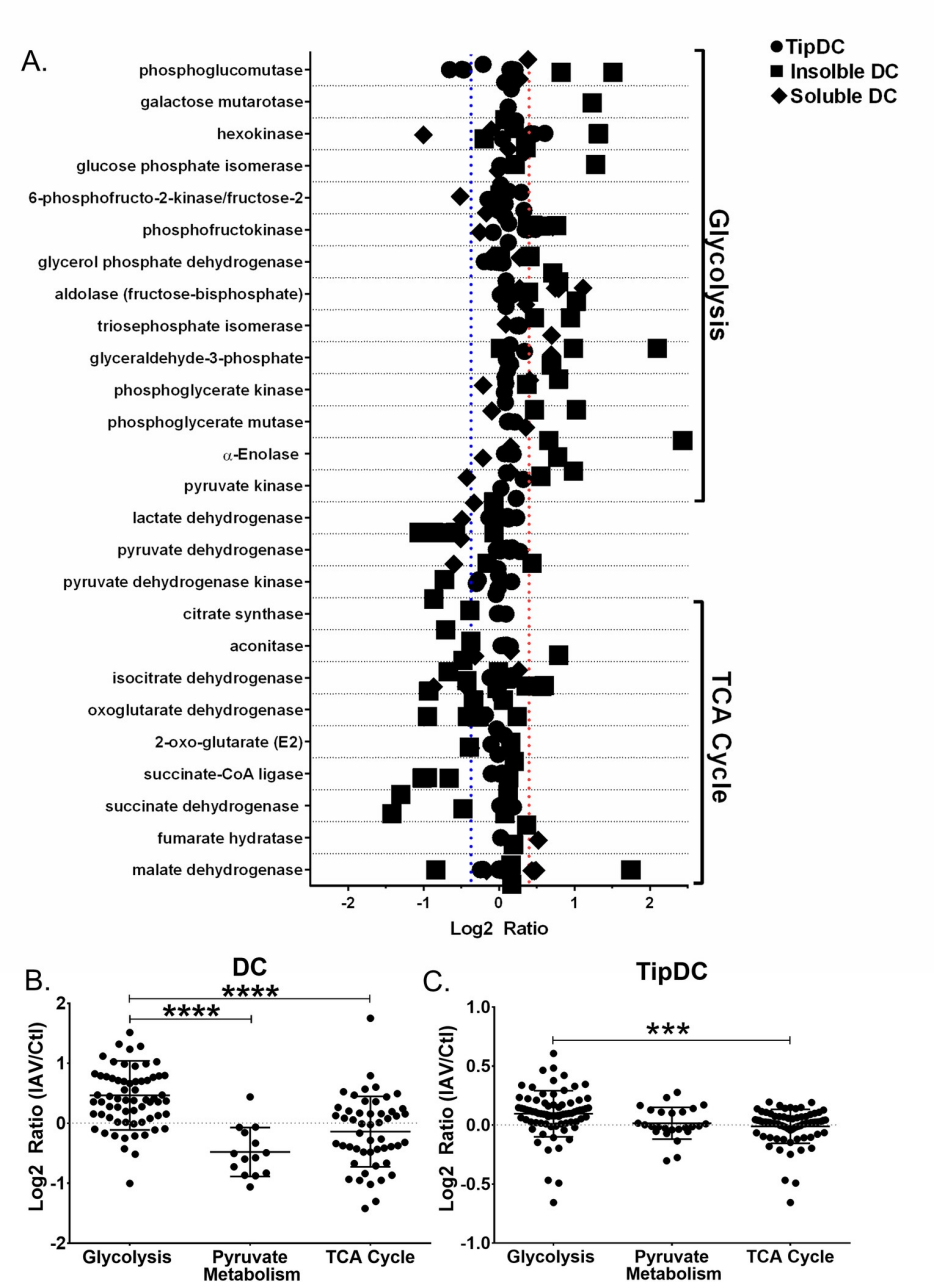
Dendritic cells globally reprogram metabolism in response to IAV infection. Proteins were extracted from *in vitro* differentiated uninfected and infected DC, separated into insoluble and soluble fractions, and subjected to mass spectrometry. RNA was extracted from lung TipDC on day 0 or 9 of IAV infection and subjected to RNA-Seq. The log2 ratio (IAV/Ctl) of transcripts and peptides were determined. Identifiers were converted to gene symbols and official protein names, each dataset were manually searched for all NCBI listed genes and synonyms corresponding to the major regulatory enzymes of glycolysis, pyruvate metabolism, and TCA cycle. (A) All isoforms of each enzyme were grouped (y-axis) and log2 ratios plotted. (B) Peptides, from the *in vitro* DC proteome, of enzymes were grouped into glycolysis, pyruvate metabolism, and TCA cycle. (C)TipDC transcripts were grouped into glycolysis, pyruvate metabolism, and TCA cycle.

We found many similarities when comparing *in vitro* and *in vivo* IAV-infected DC datasets. Fig 6D depicts the increased TipDC transcripts corresponding to glycolytic proteins and most of the glycolytic proteins from *in vitro* IAV-infected DC were increased (S1D Fig). TipDC significantly increased hexokinase, phosphofructokinase, triosephosphate isomerase, phosphoglycerate kinase, and enzymes that convert pyruvate to acetyl CoA and lactate (Fig 6D). We then overlaid gene symbols identified from TipDC transcripts and *in vitro* differentiated DC proteins on the enzymes of Glycolysis, Pyruvate metabolism, and TCA cycle (Fig 7A). The glycolytic enzymes tended to have positive log2 ratios, indicating an increase with IAV infection, while the TCA cycle enzymes were skewed negatively (Fig 7A). Next we combined the transcripts from each metabolic pathway and compared them. We found the IAV to control log2 ratios of transcripts of glycolytic enzymes were significantly higher than those of the TCA cycle for *in vitro* differentiated DC (Fig 7B). Likewise, when we performed the same analysis of TipDC isolated from mouse lungs following IAV, we found a very significant difference in IAV to control log2 ratios of glycolysis versus TCA cycle enzyme transcripts (Fig 7C). In both cases, the values above zero log2 ratio reflect enzymes whose gene transcripts or protein peptides increased with IAV. Thus, irrespective of the IAV strain used *in vitro*, DC differentiation *in vitro* or *in vivo*, or infection *in vitro* or *in vivo*, we found dendritic cells globally reprogram metabolism in response to influenza infection.

## Discussion

Our data indicate dendritic cells alter their metabolism in response to IAV infection similar but distinct from our previous findings in human primary respiratory epithelial cells [1]. Unlike our previous observation in epithelial cells, the only significant metabolic difference (p = 0.0102) in DC infected with replicating and non-replicating virus was lowered proton leak (Fig 3C and S3C Fig). This finding is reasonable given that IAV non-productively infects DC, whereas epithelial cells are the primary source of IAV propagation in the lungs and viral propagation requires high metabolic activity [78, 96, 97]. In our previous epithelial study, we found glycolysis was significantly lower in the absence of viral replication, and epithelial cells infected with replicating IAV lack metabolic flexibility and die rather than adapt to changes in available nutrients [1]. In contrast, here we found IAV infection, irrespective of BPL inactivation, increased DC glycolysis as well as capacity and flexibility for fueling respiration without loss of viability in substrate limiting conditions (S4C Fig). These data reinforce the idea that metabolic reprogramming of parasitized cells reflects aberrant changes driven by viral replication while infected dendritic cells harbor the virus while maintaining metabolic control. However, we found processes, such as non-glycolytic extracellular acidification, non-mitochondrial oxygen consumption, and glucose uptake, were significantly lower when IAV was BPL inactivated. These differences in DC transduction of signals that govern metabolic substrate import and product export may be worthy of closer examination.

Viral infections, like those caused by IAV, have devastating consequences for cancer patients, who often receive metabolic modulators in their treatment regimens that have the potential to alter immune function. Our current findings, in the context of our previous work, indicate the effects of these treatments may be cell and pathogen specific. Indeed, in the past 10 years, there has been considerable focus on shared pathways in T lymphocytes and cancer metabolism, and more recently, it has become clear that APC metabolism can also be altered by activation with TLR agonists PMA and LPS [14, 16–19, 41, 98, 99]. However, the metabolic demands of innate immune cells in response to IAV infection or IAV-associated TLR activation were unclear. Based on the work of pioneers in this field, we anticipated IAV and LPS would have stark differences in bioenergetics [18, 19, 59, 61, 100]. Indeed, when we compared IAV-infected DC to LPS-activated DC, we found basal respiration, ATP production, maximal respiration, spare respiratory capacity, and coupling efficiency were significantly different (S3C Fig). The most striking difference was in glycolytic ATP production, where IAV-infected DC produced an average of 393.5 pmol/min/RFU 95%CI [259.9, 527.1] more ATP than LPS-treated DC (Figs 3D and S3D). Using both healthy controls and LPS as reference points allowed us to compare a spectrum of mitochondrial fitness and determine that IAV-infected DC retain respiratory function based on their FCCP response in the mitochondrial stress test (Fig 3C). Moreover, although the proportion of ATP produced from OXPHOS was significantly lower than that produced by glycolysis, mitochondrial ATP production upon IAV infection was reduced by only 20% in DC. Thus, unlike LPS treatment, IAV infection did not severely impair respiration. We were surprised to find DC stimulated with PolyIC or R848, induced significantly different respiration, mitochondrial ATP production, spare respiratory capacity and glycolytic ATP production than IAV infected DC (S3 Fig). These TLR agonists are common models of IAV and overlap in signaling pathways, interestingly, they had a very different response to FCCP. The latter was confirmed with an FCCP-independent ATP rate assay that showed R848 produced 57.47 pmol/min/RFU 95%CI [-75.56, -39.38] less mitochondrial ATP than PolyIC (p < 0.0001) even though both agonists increased DC glycolysis similarly (Figs 3A, 3D and S3D). Thus, we have highlighted an additional layer of complexity in the mechanisms underlying activated DC metabolic rewiring and found that TLR agonists and IAV infections induce unique bioenergetics. Future studies in this area will better dissect these signaling pathways and tease apart how they orchestrate metabolic reprogramming.

Using quantitative proteomics and transcriptomics, we found global rewiring of metabolism early in the DC response to IAV infection *in vitro* and in TipDC 9 days following IAV infection *in vivo*. This finding was validated and expanded on by measuring metabolism of specific substrates, extracellular and intracellular metabolite concentrations, substrate product and flux rates for glycolysis and respiration, and bioenergetic functional assays of ATP production or metabolic capacity and flexibility. Combined, these observations delineate the central features of the metabolic response of DC to IAV infection, showing a significant increase in glycolysis and glycolytic ATP production that is decoupled from canonical pyruvate oxidation in the TCA cycle in favor of utilizing glutamine. This phenomenon is reminiscent of Warburg metabolism, a phenotype that reflects an increase in demand for anabolic metabolites and the energy to assemble them into macromolecules necessary for cancer cell proliferation as well as proliferation and effector functions of adaptive immune cells [14, 15, 39–42, 83, 87, 99, 101]. It has long been appreciated that DC motility from the lungs to draining lymph nodes is critical to mounting an effective immune response to IAV, and motility is an energy-demanding process that is directly coupled to ATP availability [78, 90, 102, 103]. We observed a dramatic increase in DC motility following IAV infection and found IAV-infected DC derive approximately 17 times more ATP from glycolysis than mitochondrial OXPHOS, corresponding to 10 fmol/min versus 0.6 fmole/min per infected DC (Figs 5A and 3D). One intriguing possibility is this pool of glycolytic ATP is critical for infected DC trafficking. In support of this, we found inhibiting c-Myc abolished IAV-induced glycolysis and DC motility (Figs 4B and 5A). Given IAV-infected DC have increased capacity to utilize diverse substrates and the flexibility to toggle between them, we are currently unable to fully explain why inhibiting mitochondrial oxidation of specific substrates would diminish motility or affect priming of T cells (Figs 2 and 5). This result may indicate metabolic compensation by DC involving use of suboptimal mitochondrial fuels comes at a cost that weakens their effector function.

IAV-infected DC appear to acquire a high level of plasticity in energy substrate metabolism, including greater carbon and energy source flexibility. Indeed, decoupling of glycolysis and TCA cycle in cancers was once considered a mitochondrial defect but is now postulated to produce a more robust and flexible metabolic program [43, 104]. c-Myc is considered a master regulator of cancer metabolism, and metabolic reprogramming by c-Myc is thought to allow cancer and activated immune cells to sustain supplies of anabolic building blocks while generating energy for their assembly [14, 63, 64, 67, 71]. Our data supports applying this idea to IAV-infected DC, given we found c-Myc expression increased in response to IAV infection and c-Myc inhibition significantly reduced glycolysis, respiration, and glutamine import while preventing IAV-induced DC motility and induction of CD25+ and CD44+ CD8 T cells (Figs 4, 5A–5F and S5E). Nevertheless, as DC mature, they downregulate expression of c-Myc and n-Myc while maintaining constant expression of l-Myc even after activation [67, 68]. Paradoxically, mice with l-Myc-deficient DC have a reduced T cell response during bacterial or viral infection [67, 68]. In this respect, although it is possible that our reagents were nonspecific, we suggest this explanation is unlikely for several reasons: the primers we used are specific for c-Myc [105], these primers amplified only one species, and the c-Myc antibodies we used detected a single band of the appropriate size. Moreover, we observed a transient increase in response to activation stimuli (i.e., IAV infection) at the RNA and protein levels, whereas others report l-Myc remains constant in activated DC [68]. Consistent with this finding, we found l-Myc protein levels did not change over the first 17 hours of DC infection with IAV (DNS). Importantly, we found c-Myc inhibitor treatment blocked or diminished critical DC immune functions. The exact cause of this effect remains unclear, but it correlates with a loss of the DC glycolytic response to IAV. With respect to the interplay of the Myc paralogs and determining their specific roles in relationship to DC stimuli, phenotype, and pathogen response are open questions [68, 106] that now include their specific role in IAV infection.

c-Myc directly regulates glutaminase, transcriptionally activates glutamine transporters and promotes glutamine metabolism including glutamine catabolism to glutamic acid for anaplerosis and NADPH and in cancer, transcriptionally coordinates glutaminolysis and dependence on exogenous glutamine that cannot be rescued by adding TCA intermediates [63, 65, 107–112]. In cancer, glutamine utilization confers several advantages over glycolysis coupled to TCA cycle. This is primarily because during the conversion of glutamate to α-ketoglutarate and the following steps, normal levels of reducing equivalents are produced independently of Acetyl CoA, glycolysis, and FAO while supplying ample building blocks for purine nucleoside biosynthesis and *de novo* protein and fatty acid synthesis. Thus, glutamine metabolism has the added benefit of increasing proton buffering capacity and cellular redox homeostasis and glutaminolysis inhibition increases ROS [70, 113, 114]. Meanwhile, the first step of the TCA cycle is sensitive to ROS [64, 115–117] as is the ETC and membrane permeability, and this mitochondrial damage potentiates ROS [118, 119]. Hence, glutamine oxidation may be advantageous for activated innate immune cell that produces ROS in conjunction with other effector functions that require high anabolism and energy production. This idea is consistent with our observations that infected DC redirect a large portion of pyruvate away from the TCA cycle while simultaneously increasing glutaminolysis, capacity for oxidizing glutamine and glutamine import. These changes will significantly deplete intracellular glutamine concentrations and α-ketoglutarate concentrations if the mitochondrial pyruvate carrier is inhibited and c-Myc is increased (Figs 2C, 2D, 4A, and Tab E S2 File). Recently, sustained import and utilization of glucose and glutamine were shown to stabilize c-Myc and were required for T cell renewal and clonal expansion [120]. Determining the mechanisms of glutamine utilization in DC will advance our understanding of their basic metabolism and will lead to a clearer picture of immune function in the IAV-infected lung microenvironment.

In conclusion, our findings support the view that dendritic cells specifically rewire their metabolism based on activation stimuli or IAV infection, this may be controlled by c-Myc, and is required for optimal motility and T cell priming. This finding is significant, given reducing DC migration after viral infection profoundly reduces the amplitude of the T cell response and the strength of antigenic stimulation regulates T cell progression [90, 121]. Whether these restraints on DC metabolism result in a defect in antigen capture, processing, or MHC loading/presentation or if long term quantity or quality of expanded virus-specific T cells is impacted remains unclear. Importantly, it appears dendritic cells orchestrate coordinated bioenergetic and metabolic changes in response to IAV infection irrespective of strain and are similar regardless of DC origin or infection model (i.e. *in vitro* differentiated or lung TipDC *in vivo*). Although incomplete, these data suggest the net result of blocking metabolic reprogramming therapeutically in patients or limiting metabolites *in situ* could have detrimental consequences in terms of mounting an appropriate immune response. Moreover, it is also tempting to postulate that local metabolite depletion in the lung may act to limit lung injury by placing a governor on the immune response. However, the metabolite milieu of the IAV-infected lung and lung-lining fluid has not been well established, and this limitation must be addressed to determine if variability in metabolite concentrations impact cellular responses or secondary infections. Additionally, it is unknown if metabolic reprogramming occurs in humans with community-acquired respiratory viral infections and if so if it can be exploited therapeutically to enhance or reduce the immune response to IAV. These findings extend our understanding of immune metabolism into influenza infection of a critical cell type that bridges innate to adaptive immunity and establishes an appropriate *in vitro* model to study this metabolic phenomenon.

## Materials and methods

### Ethics statement

All experiments using mice were performed in compliance with the Guidelines of Care and Use of Laboratory Animals and approved by University of Tennessee Health Science Center (UTHSC) Animal Care and Use Committee. Experiments were conducted under IACUC protocol # 16–138.0.

### Tissue culture and mouse work

#### Bone marrow-derived dendritic cells (DC)

Female C57BL/6J (B6) mice were purchased from The Jackson Laboratory (Bar Harbor, ME) and were maintained in specific-pathogen-free facilities at UTHSC, Memphis, TN. Primary bone marrow-derived DC were isolated and differentiated (104). Briefly, after the epiphysis bone marrow was flushed out, red blood cells were lysed, cell viability was determined with AO/PI, and 2 million viable precursors per 100 cm^2^ polystyrene plate were seeded in 10 ml RPMI supplemented with penicillin (100 U/ml), streptomycin (100 μg/ml), fetal bovine serum (FBS, 10% v/v) (Gibco Grand Island, NY) and 20 ng GM-CSF (R&D systems, Minneapolis, MN) per ml medium. Medium containing 20 ng GM-CSF/ml was supplemented on days 3, 6, and 8. DC were harvested on day 10.

#### T cell isolation

Mice were anesthetized with 2,2,2-tribromoethanol (Sigma-Aldrich, St. Louis, MO) with a dose adjusted to the individual body weight and infected intra-nasally with 10^4^ LD_50_ of PR8 virus in 30 μl sterile phosphate-buffered saline (PBS) (PMID:25668410, PMID:23388712). Weight loss and survival of infected mice were monitored daily over 21 days. Mice were sacrificed, and spleens were harvested from virus-challenged (primed) or naïve 8–12 week-old C57BL/6 or C57BL/6-Tg(TcraTcrb)1100Mjb/J (OT1) male and female mice. T cells from challenged mice were used for flow cytometry experiments only. Spleens were manually disrupted by grinding organ tissue between the frosted ends of two sterile glass microscope slides in sterile PBS containing 2% FBS (PMID: 29293541). T cells were isolated by magnetic separation using EasySep^TM^ Mouse CD8+ T Cell Isolation Kit with anti-CD8 magnetic beads (StemCell^TM^ Technologies, Vancouver, Canada) (purity>99%) for CD8+T Cells or EasySep Mouse T Cell Isolation Kit (StemCell Technologies) for T cell isolation by negative selection.

#### Intranasal infection and lymphocyte isolation

Female and male 6–8 week-old C57BL/6 mice were purchased from The Jackson Laboratory. All mice were acclimated for two weeks prior to inoculation with IAV. All infections were conducted in an BSL2 facility where animals were assessed daily. Mice with severe morbidity (greater than 30% weight loss plus severe clinical impairment) were humanely euthanized according to our approved protocol. Prior to intranasal delivery of 30 μl virus diluted at EID_50_ 2000, the mice were anesthetized with intraperitoneal injection of 2,2,2-tribromoethanol (Avertin). Throughout the course of the infection, mice were monitored by daily weighing and assessment of clinical distress symptoms (e.g., ruffled fur, hunched back, lethargy). At day 0 or 9, mice were euthanized, and the lungs were collected after perfusion *in situ* via the ventricles with 40–50 ml PBS. The lungs were rinsed with PBS, minced with fine scissors, and gently pushed through a 40-μm strainer with a syringe plunger. The suspension was then washed with Click’s medium and incubated in digestion buffer [Click’s plus 50 U/ml collagenase IV and 0.001% DNase I (Sigma-Aldrich)] for 30–90 minutes at 37°C on a rocking platform. Lungs tissue was passed through a 1 μm sieve. Cells were collected, washed (40 ml Click’s medium), and resuspended in sterile 2% PBS. Lymphocytes were isolated with Percoll density gradient.

#### Virus propagation and titer

A/PuertoRico/8/34 virus (IAV in the text) is a mouse adapted highly pathogenic influenza virus (PR8) [122]. This virus was cultured in pathogen-free antibiotic-treated eggs (generously provided by Dr. Paul Thomas) that were inoculated on day 9 in bulk from a single viral stock. Allantoic fluid was harvested 48 hours following inoculation and aliquoted into 50 ml conical tubes and stored at -80°C. Individual bulk viral stock was then thawed, aliquoted, and stored at -80°C; titer was also determined. Tissue culture infectious dose at 50% (TCID_50_) was then determined by serial dilution in near confluent Madin-Darby canine kidney (MDCK) cells [purchased from American Type Culture Collection (ATCC CCL-34)] in the presence of 1 mg/ml trypsin (Sigma-Aldrich), verified with 50% egg infectious doses (EID_50_), and calculated according to the Reed and Muench method [123]. A/PuertoRico/8/34 virus was added at MOI of 5 for 2 hours to DC. Virus-laden medium or blank infection medium was then removed, and the infection proceeded for indicated times.

### Proteomics: sample preparation, mass spectrometry, and data processing

We have previously described these proteomics methods elsewhere in detail. Details regarding sample acquisition, MS parameters, and data processing can be found in Smallwood et al 2011 [124]. Details specific to quantitative proteomics using isobaric tags can be found in Smallwood et al 2017 [1]. For details specific to quantitative proteomics using stable isotopically labeled proteomics, see Lopez-Ferrer et al 2009 [23]. Brief summaries of pertinent details are also provided below.

#### Sample preparation

DC were infected for 17 hours with PR8 (MOI 5 or 1) followed by lysis, homogenization, and fractionation by centrifugation. iTRAQ samples were digested through tryptic digestion by using a FASP Protein Digestion Kit (Protein Discovery, San Diego, CA) according to the manufacturer’s instructions with slight modifications (Supplemental Data). Samples were desalted by C18 solid-phase extraction (SPE) before isobaric labeling (SUPELCO). Digested samples were then processed according to the manufacturer’s directions for iTRAQ 4-plex labeling (AB Sciex, Framingham, MA). A separate set of DC samples was processed by performing trypsin-catalyzed ^18^O labeling. Before high pH reverse-phase fractionation with concatenated pooling, samples were desalted by C^18^ SPE (SUPELCO). All samples were processed in a custom liquid chromatography system using reversed-phase C^18^ columns. The iTRAQ samples were analyzed by using a Velos Orbitrap mass spectrometer (Thermo Scientific, Waltham, MA), and ^18^O-labeled samples were analyzed by using an LTQ-Orbitrap mass spectrometer (Thermo Scientific). Both systems were equipped with custom ion funnel–based atmospheric pressure ionization sources and electrospray ionization interfaces.

#### Data processing

Raw files were compared with a concatenated NCBI *Mus musculus* database and contaminant database by using SEQUEST v.27 (rev. 12). The resulting sequence identifications were rescored by using MS-GF and filtered to a 1% false-discovery rate by using the target-decoy approach and MS-GF–derived spectral probabilities. Reporter-ion intensities were quantified by using the MASIC tool (2). Missing reporter ion channel results were excluded from analysis. Redundant peptide identification reporter ions were summed across fractions, and median central tendency normalization was used to account for channel bias and then log_2_-transformed. Soluble and insoluble fractions were analyzed separately by using DanteR software. The ANOVA model included treatment and peptide effects. The remaining treatment effect and p-value were calculated by using Student’s *t*-test. Benjamin-Hochberg multiple-testing error correction was applied to the results of this hypothesis testing, and proteins with a p-value ≤0.05 were considered significant.

#### Bioinformatics

Peptide identifiers were converted to ENSEMBL and ENTREZID gene identifiers and protein names with DAVID. The log2 ratio of significant peptides was converted to fold change, and the soluble and insoluble proteins were separated by fold change (i.e., increasing and decreasing ≥2-fold). iTRAQ and SIL proteomes were combined, and redundancies were removed by selecting the most significant peptide. The ≥2-fold protein lists were then submitted to DAVID for enrichment analysis [24]. KO pathways were cross referenced with KO Class and subcategory. We obtained a network summary of localization changes in confidently identified proteins with significant enrichment in metabolic pathways was performed as previously described [25]. Briefly, subclasses of KO metabolic proteins were organized based on location in the soluble or insoluble fraction and connected to protein nodes with red or blue edges based on increasing or decreasing abundance, respectively. Protein nodes were then colored by KEGG subclass: carbohydrate metabolism (red), amino acids metabolism (orange), nucleotide metabolism (purple), energy metabolism (green), lipid metabolism (yellow), and glycan biosynthesis and metabolism (blue). A Cytoscape network was built essentially as previously described [125]. Briefly, we submitted both the soluble and insoluble SIL proteomes to DAVID to select proteins in the KEGG glycolysis pathway. We used PPI spider to determine the glycolytic protein-protein interaction network [126]. Then, we mapped the interaction network to the glycolytic network and used Cytoscape to overlay abundance data from the proteomes.

### Transcriptomics: sample preparation, RNA-Seq, and data processing

#### Sample preparation

RNA was extracted from sorted TipDC using 1 mL TRIzol reagent (Invitrogen, Carlsbad, CA). RNA was extracted from TRIzol suspensions using the Zymo Research Direct-zol RNA miniprep kit (Zymo, Irvine, CA), treated with 10 U DNAse for 20 min at RT, and then concentrated using the Qiagen RNeasy MinElute clean up kit (Qiagen, Hilden, Germany). Purified RNA quality was checked using the Nano Total RNA kit on an Agilent 2100 Bioanalyzer (Agilent, Santa Clara, CA).

#### RNA-Seq

We used Illumina’s TruSeq RNA v2 sample preparation protocol according to manufacturer’s instructions for generation of RNA-Seq libraries. Briefly, this protocol involved the following steps: A. cDNA Synthesis: mRNA was purified via oligo(dT) beads followed by fragmentation using divalent cations and heat. The 1st strand cDNA synthesis was performed using random primers, followed by 2nd strand cDNA synthesis. B. cDNA library preparation: cDNA fragments were DNA blunt end repaired followed by 3' adenylation of DNA fragments. Sequencing adapters were then ligated to the ends utilizing T-A pairing of adapter and DNA 2 fragments. This step was followed by PCR amplification of the library. The cDNA libraries were run on an Illumina HiSeq2000.

#### Data processing

Data quality was checked with fastqc software. Paired end RNA-Seq data were aligned to the Mouse mm10 genome using TopHat (with Bowtie2). Only concordant pairs with mapping quality > 10 were kept. The number of reads assigned to each gene was found using Bioconductor R package. Count data were analyzed using edgeR Bioconductor package (GLM formulation). Before this procedure, genes without > = 3 counts in every sample for at least one group were filtered out. Genes were declared differentially expressed if they had FDR < 0.05 and log2 FC > 1. Multidimensional scaling (MDS) as implemented in EdgeR function plotMDS (in two dimensions) was used for data visualization. Briefly, MDS takes as input the set of pairwise distances between any two samples and plots points in 2-dimensional space (plane), attempting to preserve the original distances between any two samples as much as possible.

#### Bioinformatics

Transcript identifiers were converted to official gene symbols with DAVID and linked to expression levels in Excel (Microsoft, Redmond, WA). The XLSTAT OMICS data analytics package was used for generating the heat map and expression analysis. Differential expression was determined from three day 0 controls and four day 9 IAV-infected samples. The standard deviation threshold for this analysis was 50%, eliminating 6,643 transcripts out of 13,285. Significant differences in expression were selected based on p-values generated from a parametric comparison of differences using Tukey’s honest significant difference test for multiple comparisons with Benjamini-Hochberg post hoc corrections.

### Metabolic assays

#### Metabolite depletion–Live/dead viability assay

DC were cultured as described above. After 24 hours of adherence, RPMI 1640 complete medium was replaced with fresh RPMI 1640 complete medium or RPMI metabolite-depleted medium for 3 hours (i.e., RPMI without glucose or glutamine or RPMI without both). Next, the cells were treated as above in complete or depleted media, followed by infection for 17 hours. The cells were labeled with Calcein-AM (2 μM) and EthD-1 (4 μM) and incubated for 20 minutes at room temperature. Cell viability was measured using a Live/Dead Viability/Cytotoxicity assay kit for mammalian cells (Thermo Scientific) with a fluorescent plate reader at 494/517nm and 528/617nm for Calcein-AM and EthD, respectively.

#### Metabolic flux

Glycolytic flux was determined by measuring the detritiation of [3-^3^H]glucose (Sigma-Aldrich) as previously described [127]. Fatty acid beta-oxidation flux was determined by measuring the detritiation of [9,10-^3^H]-palmitic acid (Sigma-Aldrich) [128]. Glutamine oxidation flux was determined by calculating the rate of ^14^CO_2_–release from [U-^14^C]-glutamine (Sigma-Aldrich) [129]. Pyruvate oxidation flux was determined by calculating the rate of ^14^CO_2_–release from [2-^14^C]-pyruvate (Sigma-Aldrich) [130]. Glucose oxidation flux through the pentose phosphate pathway (PPP) was determined by calculating the rate of ^14^CO_2_–release from [1-^14^C]-glucose (Sigma-Aldrich), as previously described [14, 131]. The concentration of free fatty acids (≥C8) was determined using the Free Fatty Acid Assay (Abcam, Cambridge, MA) with a coupled enzyme assay, a fluorometric assay with the fluorescent emission/excitation wavelengths 535/587 nm. The resulting fluorometric product was proportional to the free fatty acids present and quantifiable with the palmitic acid standards.

#### Glucose and glutamine quantification

DC were treated as above, and after 17 hours, cells and supernatants were collected alongside blank medium as controls. Extracellular glucose was quantified with a glucometer against glucose standards essential as previously described [1]. Glucose levels (mg/dl) in the blank medium were determined alongside DC +/- treatments. Glucose uptake was calculated by subtracting the glucose in the control, IAV, IAV^BPL^, PolyIC, R848, or LPS medium from the cell-free blank medium. Internal and external glutamine were quantified from supernatant and cell lysates, respectively, following the Glutamine/Glutamate Glo assay manufacturer's protocol (Promega, Madison, WI). Briefly, samples were prepared in PBS, diluted to fit in to the linear range of the glutamine or glutamate standard curve, and.25 μl sample or standard transferred to a 96 well luminescence assay plate and reaction allowed to proceed for 30 minutes at room temperature. Next, substrate detection reagent was added and incubated for an additional 60 minutes and metabolite detection was performed using a bioluminescent NADH detection method with luminescence measured using a BMG CLARIOstar microplate reader.

### Bioenergetic assays

Glycolysis converts glucose to lactate and produces one H+ per lactate. The TCA cycle reactions, that fuel electron transport and oxidative phosphorylation, consume oxygen and produce carbon dioxide. One can measure the net result of these reactions is the extracellular acidification rate (ECAR), proton production rate (PPR) and oxygen consumption rate (OCR) with a Seahorse XFp bioanalyzer (Agilent). ECAR, OCR, and PPR were measured using the XFe-96 Extracellular Flux Analyzer (Agilent).The conditions were optimized for each assay type with the final concentrations of: 10 mM glucose, 50 mM 2-DG, 1 μM oligomycin, 2 μM FCCP, and 0.5 μM antimycin/rotenone and cell density of 1.5x10^5^ cells per well The cells were seeded in poly-D-lysine-coated Seahorse XFe96 microplates at 37°C in 5% CO_2_ for 24 hours and were either left untreated (control) or infected for 17 hours at MOI of 5 using viable virus or IAV^BPL^ or treated with LPS (50 ng/ml), PolyIC (1 μg/ml), or R848 (1 μg/ml).After 17 hours, the cells were switched to XF medium which was supplemented with L-glutamine (2 mM) for Glyco stress test or with glucose (10 mM), L-glutamine (2 mM), and sodium pyruvate (1 mM) for Mito stress test and ATP assay ECAR, OCR, and PPR were monitored for 2 hours. After each run, cells per well were quantified with CyQUANT^TM^ (Thermo Scientific) to derive normalization factors. Rates were calculated using the Seahorse XF real time report generator and imported to GraphPad Prism (GraphPad, San Diego, CA) for statistical analysis.

#### Glyco and mito stress test with c-Myc inhibitor

DC were seeded as described above and left untreated (control); pretreated with c-Myc Inhibitor (MilliporeSigma, Burlington, MA) for 4 hours at 0.5 μM, 1.0 μM, or 2.0 μM; and infected for 17 hours at MOI of 5 with PR8 viable virus. We followed the assay protocols for the Mitochondrial Stress Test and the Glycolytic Stress Test from Agilent and used empirically determined drug concentrations listed above per manufactures recommendations to titrated drugs prior to assays.

#### Mito fuel flex test

DC were treated as described above. Calculations were performed with the Seahorse Mito Fuel Flex Test Report Generator plug in for Excel (Microsoft), and inhibitor injections followed manufacturer protocols using sequential or combined injections of 4 μM etomoxir, 3 μM BPTES, and 2 μM UK5099. Targets and calculations are briefly summarized below:

**Table:** 

Test	Injection 1	Injection 2
Glucose Dependency	UK5099	BPTES/Eto
Glutamine Dependency	BPTES	UK5099/Eto
Fatty Acid Dependency	Etomoxir	UK5099/BPTES
Glucose Capacity	BPTES/Eto	UK5099
Glutamine Capacity	UK5099/Eto	BPTES
Fatty Acid Capacity	UK5099/BPTES	Etomoxir

$$\left[\frac{Baseline\;OCR-Injection\;1\;OCR}{Baseline\;OCR-Injection\;2\;OCR}\right]*100\%$$ (1)

$$\left[1-[\frac{Baseline\;OCR-Injection\;1\;OCR}{Baseline\;OCR-Injection\;2\;OCR}]\right]*100\%$$ (2)

Dependency Eq (1), Capacity Eq (2), and Flexibility (i.e., the difference between the capacity and the dependency for each substrate) were calculated with Seahorse XF Flex Fuel Report generator and exported to GraphPad Prism for statistical analysis. The combined percent oxidation capacity or dependence is the sum of the individual values for glucose, glutamine, and fatty acids.

#### ATP rate assay

DC were seeded and left untreated (control); treated with TLR agonists or infected for 17 hours at MOI of 5 with PR8 viable virus as described above. We followed the assay protocols for the Seahorse XFp-Real-Time ATP Rate Assay from Agilent and used empirically determined drug concentrations (listed above) per manufactures recommendations to titrated drugs prior to assays. We measure ECAR, OCR, and PPR under basal conditions and after serial addition of mitochondrial inhibitors (oligomycin and rotenone/antimycin A) and calculated the total cellular ATP production rates and pathway-specific mitoATP and glycoATP production rates following the manufactures guidelines and reported post data acquisition using the Seahorse XF Real-Time ATP Rate Assay Report Generator graphed in GraphPad. Briefly, the stoichiometry of ATP, H+ and lactate in the glycolytic pathway are equal (i.e. two each from one glucose molecule)

Thus, the rate of glycolytic ATP production is equivalent to the glycolytic proton efflux rate (glycoPER) and can be calculated (Eq 3). Likewise, the OCR that is coupled to ATP production during OXPHOS can be calculated by subtracting the OCR after adding the ATP synthase inhibitor, oligomycin (Eq 4). Transformation of OCR ATP to the rate of mitochondrial ATP production consists of: multiplying by 2 to convert to oxygen atoms consumed and then multiplying by an average P/O value of 2.75 (the number of molecules of ADP phosphorylated to ATP per atom of O reduced by an electron pair flowing through the electron transfer chain). Then, with these assumptions, the rate of mitochondrial ATP production (Eq 5) and the total cellular ATP Production Rate (Eq 6) are calculated.

$$glycoATP\;Production\;Rate\;\left(pmol\frac{ATP}{min}\right)=glycoPER\;(pmol\;H+/min)$$ (3)

$$OCRATP\;\left(pmol\frac{O2}{min}\right)=OCR\;\left(pmol\frac{O2}{min}\right)-OCROligo\;\left(pmol\frac{O2}{min}\right)$$ (4)

$$mitoATP\;Production\;Rate\;\left(pmol\frac{ATP}{min}\right)=OCRATP\;\left(pmol\frac{O2}{min}\right)*\;2\;\left(pmol\frac{O}{pmol}O2\right)*\frac{P}{O}\left(pmol\frac{ATP}{pmol}O\right)$$ (5)

$$ATP\;Production\;Rate\;\left(pmol\frac{ATP}{min}\right)=glycoATP\;Production\;Rate\;\left(pmol\frac{ATP}{min}\right)+mitoATP\;Production\;Rate\;\left(pmol\frac{ATP}{min}\right)$$ (6)

### Functional assays

DC were untreated (control) or pretreated with 2 μM cMyc inhibitor (MI), 3 μM Bis-2-(5-phenylacetamido-1,3,4-thiadiazol-2-yl)ethyl sulfide (BPTES), 2 μM UK5099, or 4 μM etomoxir (Eto) for 4 hours and infected for 17 hours with IAV at MOI of 5. Fresh T cells (purified as described above) were added to DC at a 1:5 ratio and incubated for 24 and 48 hours.

#### Cell motility assay

DC were seeded on Poly-D-lysine 100 μg/ml (Sigma Aldrich) precoated Oris 96-well motility plates (Platypus Technologies) at 1x10^5^ cells per well with silicon stoppers in place. Cells were maintained at 37°C in a humidified incubator with 5% CO2 overnight. After attachment, stoppers were removed, and growth media replaced. DC cells were treated with c-c-Myc inhibitor (2 μM), BPTES (3 μM), etomoxir (4 μM), or UK5099 (2 μM) and infected with PR8 for up to 17 hours at MOI of 5. Following incubation, the cells were rinsed with PBS and labelled with Calcein-AM for 20 minutes at 37°C from a Live/Dead Viability/Cytotoxicity kit for mammalian cells (Thermo Scientific). After staining, the plate was read on a BMG CLARIOstar plate reader with FITC excitation/emission filter 485/528 nm. The combined effects of motility and stimulation were evaluated by comparing the fluorescent signal detected in the non-infected wells to the signal in the virus-infected wells. Data tables from multiple experiments were combined and plotted in GraphPad Prism.

#### FITC-dextran phagocytosis

Cellular uptake of FITC-dextran was determined [132]. DC were left untreated (control) or pretreated with c-Myc (2 μM), BPTES (3 μM), UK5099 (2 μM), and etomoxir (4 μM). The DC were then infected for 17 hours at MOI of 5 with PR8 viable virus and LPS (50 ng/ml) as positive control. After 17 hours of infection, cells were labelled with 1 mg/ml FITC-Dextran 40S (Sigma-Aldrich) and incubated for 1 hour at 37°C and 4°C, respectively. Uptake was terminated by washing the cells 3x with ice-cold PBS. Cells were resuspended in fluorescence-activated cell sorting (FACS) buffer (PBS containing 2% bovine serum albumin and 0.1% sodium azide). FITC fluorescence in cells was measured using a Sony SH800 cell sorter (Sony, Japan). Fluorescence values are reported as the mean fluorescence intensity (MFI).

### Flow cytometry

FACS analysis was performed using standard methodology. Briefly, after Fc blocking, 1 × 10^6^ cells were stained with appropriate antibodies for 30 min at 4°C and then washed with PBS/2% FBS. The cells were stained in FACS buffer (PBS containing 0.5% bovine serum albumin and 0.05% sodium azide) with fluorescently labelled antibodies from BioLegend (San Diego, CA; CD11c-APC/Cy7, CD11b-BV421, CD40-FITC, CD80-APC, CD86-PE, CD8-PE/Cy7, CD4-PerCp/Cy5.5, CD69-PE, CD25-APC, CD3-BV421, CD44-BD427, CD45.1-FITC, CD3-PerCp/Cy5.5, and CD44-FITC) or Tonbo Biosciences (San Diego, CA; HLA-DR-PerCp/Cy5.5) to identify DC and T cells. The percentage for each marker was determined by flow cytometry on a Sony SH800 cell sorter, and data were analyzed using FlowJo software (Tree Star, San Carlos, CA). Fluorescence minus one (FMO) control for each marker was used to identify gating boundaries. DC responses were examined by flow cytometry after staining with anti-CD11c, MHCII, CD8, CD4, CD11b, GR-1, CD103, CD80, and CD86 antibodies. TipDC were sorted using CD11b^hi^, Ly6c/GR-1^hi^, MHCII ^hi^ DC surface marker staining on a FACS Aria cell sorter (BD Biosciences, Franklin Lakes, NJ).

#### T cell activation assay

CD8+ T cells were isolated and co-cultured with drug treated and/or infected DC at a 1:5 ratio (DC/T cell) for 24 hours at 37°C with 5% CO2. ECAR was measured to detect T cell activation in real time using anti-CD3/CD28-coated DynaBeads (Life Technologies, Carlsbad, CA) on Xfe96 (Agilent) [83–86]. ECAR was measured in unbuffered RPMI without phenol red and supplemented with final concentrations of glucose (10mM), L-glutamine (2 mM), and sodium pyruvate (1 mM) per well. Three rate measurements were performed to establish basal rate followed by 15 cycles of real-time measurements of ECAR. Data files were converted to Excel files using Wave 2.4.1. (Agilent). Data tables were combined from multiples experiments and plotted and analyzed in GraphPad Prism.

### RNA and protein assays

#### Quantitative real-time PCR

RNA was isolated from cultured DC and or treated with c-Myc for 4, 8 and 17 hours by using a Direct-zol RNA MiniPrep Kit (Genesee Scientific) per the manufacturer’s instructions. cDNA synthesis was performed using an iScript^TM^ cDNA Synthesis Kit (Bio-Rad, Hercules, CA) per the manufacturer’s instructions. The cDNA synthesis used 7 μl reaction mix consisting of 4 μl 5× iScript reaction mix and 1 μl iScript reverse transcriptase, with the remainder nuclease-free H_2_O. This mixture was incubated at 25°C for 5 minutes, 42°C for 30 minutes, and 85°C for 5 minutes. Real time RT-PCR was performed using the SYBR^TM^ Green Master Mix (Applied Biosystems, Foster City, CA). Simple relative quantification of target gene expression normalized to β-actin was performed using the 2^−ΔΔCt^ method [133]. The following primers were used: c-Myc, forward primer: 5′- CGGACACACAACGTCTTGGAA-3′ and cMyc, reverse primer: 5′- AGGATGTAGGCGGTGGCTTTT-3′. The primers were purchased from Integrated DNA Technologies (Coralville, IA).

#### Immunoblotting

Cells were rinsed and pelleted, and the pellets were homogenized on ice in a cold Tris-EDTA+0.1% NP-40 lysis buffer. Lysates (15 μg) were reduced in NuPAGE reducing reagent (Invitrogen) and boiled for 5 min prior to loading in 4–12% NuPAGE Bis-Tris Gel for separation by electrophoresis. Proteins were transferred to nitrocellulose membranes and blocked with 3% bovine serum albumin (Thermo Scientific) in 1X TBST for 1 hour. The membrane was incubated with c-Myc overnight at 4°C followed by washing and incubation with horseradish peroxidase (HRP) secondary antibody. The protein bands were detected using ECL Plus (Amersham, GE Healthcare, Chicago, IL) on Amersham Imager 600 (GE Healthcare).

## Supporting information

S1 Fig Validation and experimental design of proteomics for IAV-infected DC. DC were left untreated (Ctl) or infected for 17 hours at MOI 5 with viable virus (IAV). (A) DC were also infected for 17 hours at MOI 5 with β-propiolactone inactivated virus (IAV^BPL^). DC were fixed and stained for DAPI, influenza nuclear protein or murine α-enolase protein and visualized with confocal microscopy. (B) Control uninfected cells or IAV-infected DC (MOI 5 pfu for 17 hours) were separated into soluble and insoluble fractions. The iTRAQ labeled samples were subjected to FASP digestion, while the SIL samples received trypsin-catalyzed ^18^O/^16^O labeling. The samples were desalted with C18 SPE, processed with a custom RPLC system and analyzed with a Velos Orbitrap mass spectrometer (iTRAQ) or LTQ-Orbitrap (SIL). (C) Venn-diagram depicting the distinct total number of proteins identified by iTRAQ and SIL, as well as the overlapping number of proteins. Venn-diagram illustrating the significant number of proteins identified by iTRAQ and SIL in soluble and insoluble fractions that were upregulated and downregulated as well as the overlapping number of proteins. (D) Both soluble and insoluble SIL DC proteomes were submitted to DAVID and PPI spider to define glycolytic protein-protein interaction networks. The glycolytic network was put into Cytoscape and integrated with quantitative data from the proteomic analysis. Alpha enolase increased in soluble and insoluble (inset) proteomes and was validated with immunoblotting revealing the monomer and dimer increased in both soluble (S) and insoluble (I) networks.
(TIF)

S2 Fig DC metabolic adaptation to fuel respiration following IAV infection. DC were left untreated (Ctl) or infected for 17 hours at MOI 5 with viable virus (IAV). (A-D) The rates of pyruvate, glutamine, or long chain fatty acids oxidation for respiration were calculated as the percentage of inhibition of oxygen consumption by UK5099, BPTES, or etomoxir, which are inhibitors of mitochondrial pyruvate carrier, glutaminase, and carnitine palmitoyltransferase 1A, respectively. Capacity for a specific substrate to drive respiratory OCR was tested by determining baseline OCR, inhibiting the 2 off target substrates determining OCR, and inhibiting import of the target metabolite. Percent capacity is one minus the baseline OCR less the off-target OCR divided by the baseline OCR less the OCR after all targets inhibited times 100. Dependency on a specific substrate was tested as above, reversing the inhibitor sequence, and the percent dependence was calculated by deducting the target OCR from the baseline and dividing by the baseline OCR less the OCR after all targets inhibited times 100. Fuel Flexibility was calculated as the difference between capacity and dependency. The average capacity of uninfected or infected DC to use either pyruvate, glutamine, or long chain fatty acids was determined. The average dependence of uninfected or infected DC on the oxidation of either pyruvate, glutamine, or long chain fatty acids was determined. The average flexibility of DC to use either pyruvate, glutamine, or long chain fatty acids was determined for uninfected or infected. (E-F) DC were pretreated with UK5099, etomoxir (ETO), or BPTES +/- IAV for 17 hours, rinsed and lysed for quantification of intracellular glutamine (E) or α-ketoglutarate activity (F). The bar graphs represent the values of 4–5 independent experiments and presented as experimental mean +/- SD p-value <0.05 (*), p-value < 0.01 (**), and p-value < 0.0001 (****). B-D show one representative experiment of 5 independent experiments with corresponding capacity, dependence, and flexibility values inset.
(TIF)

S3 Fig Confidence intervals and mean differences of bioenergetics comparing TLR agonists and IAV. (A-D) DC were infected or treated with TLA agonists lipopolysaccharide (LPS), polyinosinic polycytidylic acid (PolyIC), or Resiquimod (R848) for 17 hours followed by metabolic analysis with a Seahorse Xfe96 Flux Analyzer. (A) Glycolytic function was tested while monitoring extracellular acidification rate (ECAR) with sequential injections of glucose, oligomycin (Oligo), and 2-Deoxy-D-glucose (2-DG) indicated by arrows. (B) Glucose uptake was monitored from the medium using a standard blood glucometer with glucose standard calibration curves. (C) Mitochondrial respiration was tested while monitoring oxygen consumption rates (OCRs) with sequential injections of oligomycin (Oligo), carbonyl cyanide-p-trifluoromethoxyphenylhydrazone (FCCP), and a mixture of rotenone and antimycin A (Rot/AntA) indicated by arrows. (D) DC maximal mitochondrial ATP changes induced by oligomycin plotted against maximal ATP changes upon glucose depletion determined by respirometry using Xfe96. The graphs represent the difference of the mean values from 3–4 independent experiments (3 ≥ technical replicates) and 95% confidence intervals. Significant differences among means were found with ANOVA followed by Tukey’s honest significant difference test, validated with Dunnett’s multiple comparison tests. Dashed line appears at 1, and red circles indicate confidence intervals do not overlap.
(TIF)

S4 Fig Live to dead ratio of DC in response to infection or TLR agonists with metabolite restriction. (A&B) DC were infected with CA04 or X31 influenza virus (MOI = 5) for 17 hours followed by metabolic analysis with a Seahorse Xfe96 Flux Analyzer. (A) Glycolytic function was tested while monitoring extracellular acidification rate (ECAR) with sequential injections of glucose, oligomycin (Oligo), and 2-Deoxy-D-glucose (2-DG) indicated by arrows. (B) Mitochondrial respiration was tested while monitoring oxygen consumption rates (OCR) with sequential injections of oligomycin (Oligo), carbonyl cyanide-p-trifluoromethoxyphenylhydrazone (FCCP), and a mixture of rotenone and antimycin A (Rot/AntA) indicated by arrows. The bar graphs represent mean and error bars standard deviation. Significant differences among means was found with ANOVA followed by Tukey's honest significant difference (Tukey's HSD) method. The difference of the means are plotted with Tukey's HSD 95% confidence intervals. The red circles indicate confidence intervals do not overlap. Tukey adjusted p values are symbolized by asterisks indicating adjusted p-values (* p≤0.05, ** p≤0.001, *** p≤0.0001, and **** p<0.0001). (C) DC growth medium was replaced with depleted medium 3 hours prior to infection or agonist treatment in blank or viral laden infection medium (MOI = 5) for 2 hours followed by a return to depleted medium for 17 hours. Viable, dead, and total DC were stained with Calcein-AM, ethidium homodimer and DAPI, respectively, and we measured fluorescence intensity using a microplate reader. Then mean fluorescence intensities (MFIs) and the ratio of live to dead DC were calculated. The graphs represent the mean values of 3 independent experiments +/- SD. Significant differences among means were found with ANOVA followed by Tukey's HSD, and results are summarized using compact letter display. Groups that are significantly different have different letters.
(TIF)

S5 Fig Limiting glycolysis or mitochondrial oxidation of pyruvate, glutamine or fatty acids alters the population of infected DC. (A) DC were seeded on a precoated Oris 96-well motility plate allowed to adhere for 18+ hours followed by plug removal and infection (MOI = 5) for 17 hours. Live cells were stained with Calcein-AM, and images were acquired with EVOS. Two representative microscopy images of control and IAV motility are presented. (B-G) DC were pretreated with cMyc (2 μM), BPTES (3 μM), UK5099 (2 μM) or etomoxir (4 μM) and infected for 17 hours (MOI = 5) with IAV (solid bars) or left uninfected (open bars). (B) FITC-Dextran 40S (1mg/ml) was added and phagocytosis was terminated after 1 hour at 37°C. At 4°C, fluorescence per cell was measured using flow cytometry. (C-G) DC were stained for CD11c, CD40, CD80, CD86, MHCII surface markers and quantified by flow cytometry. We selected CD11c and MHCII positive and then gated on CD40, CD80 and CD86. (A-G) Graphs represent the mean values of 3 independent experiments with technical replicates (≥2). The error bars represent the SD. The statistical differences among means were found using ANOVA followed by Tukey's multiple comparisons test. Asterisks symbolize the adjusted p-values (* p≤0.05, ** p≤0.001, *** p≤0.0001, and **** p<0.0001).
(TIF)

S6 Fig Limiting glycolysis or mitochondrial oxidation of pyruvate, glutamine or long chain fatty acids impairs infected DC function. (C-F) CD8+ T cells were isolated by negative depletion from fresh splenocytes of homologously primed female C57BL/6 mice and co-cultured with DC at a 5:1 ratio of T cells to DC for 24 hours. Cells were fixed and stained for surface markers and quantified and compensated for using FlowJo software (2 additional independent experiments were performed on different instruments and quantified/compensated using different software. These experiments produced similar trends but were excluded due to differences in compensation methods). (C) Representative FACS plots with gating strategy. (D) Bar graphs represent the mean values of 2 independent experiments +/- SD. Statistical differences among means was found with ANOVA followed by Tukey's HSD with asterisks indicating adjusted p-values (* p≤0.05, ** p≤0.001, *** p≤0.0001, and **** p<0.0001). (E) Tukey's HSD results summarized with the circles representing the mean difference and the error bars the 95% confidence intervals. (F) Tukey's HSD results to from Fig 5D are summarized with the circles representing the mean difference and the error bars the corresponding 95% confidence intervals.
(TIF)

S7 Fig Unsupervised multivariate principal component analysis (PCA) of TipDC and volcano plot of transcripts. At zero or nine days following intranasal infection of mice with IAV at (EID_50_: 2000), the lungs were homogenized, and cells extracted. Cells were antibody stained for TipDC surface markers CD11b, Ly6c, GR-1, and MHCII. TipDC controls (Ctl) from day 0 were sorted based on high levels of CD11b, Ly6c, and GR-1. TipDC from day 9 of the IAV infection (IAV) were sorted based on high levels of CD11b, Ly6c, GR-1, and MHCII. cDNA libraries were generated from RNA, sequenced, and narrowed to confidently identified transcripts. (A) Unsupervised multivariate principal component analysis (PCA), resulting in F1 and F2 with a cumulative percent variability of 78.56%. Each circle represents a TipDC transcriptome, the open circles are control, and the solid black circles are IAV. (B) Differential expression was determined using Tukey’s HSD test for multiple comparisons with a Benjamini-Hochberg post hoc false discovery rate correction. A total of 6642 transcripts were removed with nonspecific filtering to remove transcripts that were not modulated by IAV (i.e., 50% standard deviation threshold). Log10 p-values are plotted against the Log2 ratio.
(TIF)

S1 File Significantly enriched KEGG pathways altered by influenza infection of DC. Approximately 25,000 peptides were detected with corresponding reporter ions (available in the PRIDE repository). (A) 7,520 confidently identified peptides from the stable isotopically labeled (SIL) and isobaric tagged (iTRAQ) proteomes were mapped to National Center for Biotechnology Information (NCBI) reference sequence accession numbers. (B-C) Confidently identified protein identifiers from the soluble and insoluble fractions were segregated into upregulated (B) or downregulated (C) based on 2-fold or greater abundances changes and submitted to DAVID for KEGG pathway analysis. Significantly enriched KEGG pathways are listed and gene symbols associated with NCBI accession numbers are provided along with corresponding KEGG class and subclass from the BRITE hierarchy (colored circles correspond to subclasses from Fig 1).
(XLSX)

S2 File TipDC transcriptome. At zero or nine days following intranasal infection of mice with IAV at (EID_50_: 2000,) the lungs were homogenized, and cells extracted. Cells were antibody stained for TipDC surface markers CD11b, Ly6c, GR-1, and MHCII. TipDC controls (Ctl) from day 0 were sorted based on high levels of CD11b, Ly6c, and GR-1. TipDC from day 9 of the IAV infection (IAV) were sorted based on high levels of CD11b, Ly6c, GR-1, and MHCII. cDNA libraries were generated from RNA, sequenced, and narrowed to confidently identified transcripts. (A) Transcripts differential expression was determined using Tukey’s HSD test for multiple comparisons with a Benjamini-Hochberg post hoc false discovery rate correction. (B) Transcripts that increased following IAV infection were submitted to DAVID, and significantly enriched KEGG pathways are listed with corresponding KEGG class and subclass from the BRITE hierarchy. (C) Transcripts from the KEGG metabolic classes were submitted to DAVID, and significantly enriched metabolic pathways are listed. (D) Transcripts that decreased following IAV infection were submitted to DAVID, and significantly enriched KEGG pathways are listed with corresponding KEGG class and subclass from the BRITE hierarchy. (E) Unique transcripts from the Immune Pathway and Influenza A pathway were differentially expressed, and corresponding protein names are listed under their functional category. (F) Gene symbols of the metabolic transcripts from TipDC and proteins from the in vitro differentiated DC are listed per pathway with the counts per cell type by downregulated (Down) or upregulated (Up) following IAV infection (G).
(XLSX)

S1 Table Net changes in DC proteins in response to IAV infection after 17 hours. The intensities of the isobaric tag reporter ions were quantified by using the MASIC tool with the exclusion of missing reporter-ion channels or by calculating the SIL ratio for each peptide pair after accounting for singly or doubly labeled species in the ^16^O/^18^O ratio and correcting for labeling efficiency. Then, the MS/MS data were searched and filtered by using 0.5% FDR; peptides passing the filter were quantified. Then, peptides-to-protein rollup was performed.
(DOCX)

S2 Table Infection induced changes in the relative abundance of DC metabolic proteins. Influenza virus strain A/PuertoRico/68/34 (IAV) was added to DC for 2 hours, infection medium was replaced, and the infection proceeded for 17 hours followed by cell lysis and protein extraction. Proteins were labeled with SIL or iTRAQ subjected to LC-MS/MS. Both proteomes were combined, redundancies removed, and confidently identified peptides with abundance changes of 2-fold or greater linked to protein identifiers. The lists of upregulated and downregulated proteins were submitted to the Database for Annotation, Visualization, and Integrated Discovery (DAVID) v6.7 and mapped to KEGG pathways. Significantly enriched major metabolic pathways are listed with proteins, soluble (Sol) or insoluble (Insol) fraction and fold change (FC) designated.
(DOCX)

S3 Table Proteomic coverage of the pyruvate dehydrogenase complex. The proteome was queried for components of the pyruvate dehydrogenase complex and subunits reported with corresponding functions, fold changes, names, and localization to soluble (Sol) or insoluble (Insol). A summary of identified metabolic proteins that relocalized from the soluble to insoluble fraction or vice versa is provided. Fold enrichment significance was generated from DAVID functional annotation clustering and the biological significance indicated with p-value derived from KEGG pathways. Fold changes from non-unique proteins identified in the same metabolic pathway were averaged.
(DOCX)
