# Supplementary figures and images for: Dynamic metabolic reprogramming in dendritic cells: An early response to influenza infection that is essential for effector function
Source: PLoS Pathog. 2020 Oct 26;16(10):e1008957. doi: 10.1371/journal.ppat.1008957 (PMC7707590; doi:10.1371/journal.ppat.1008957)

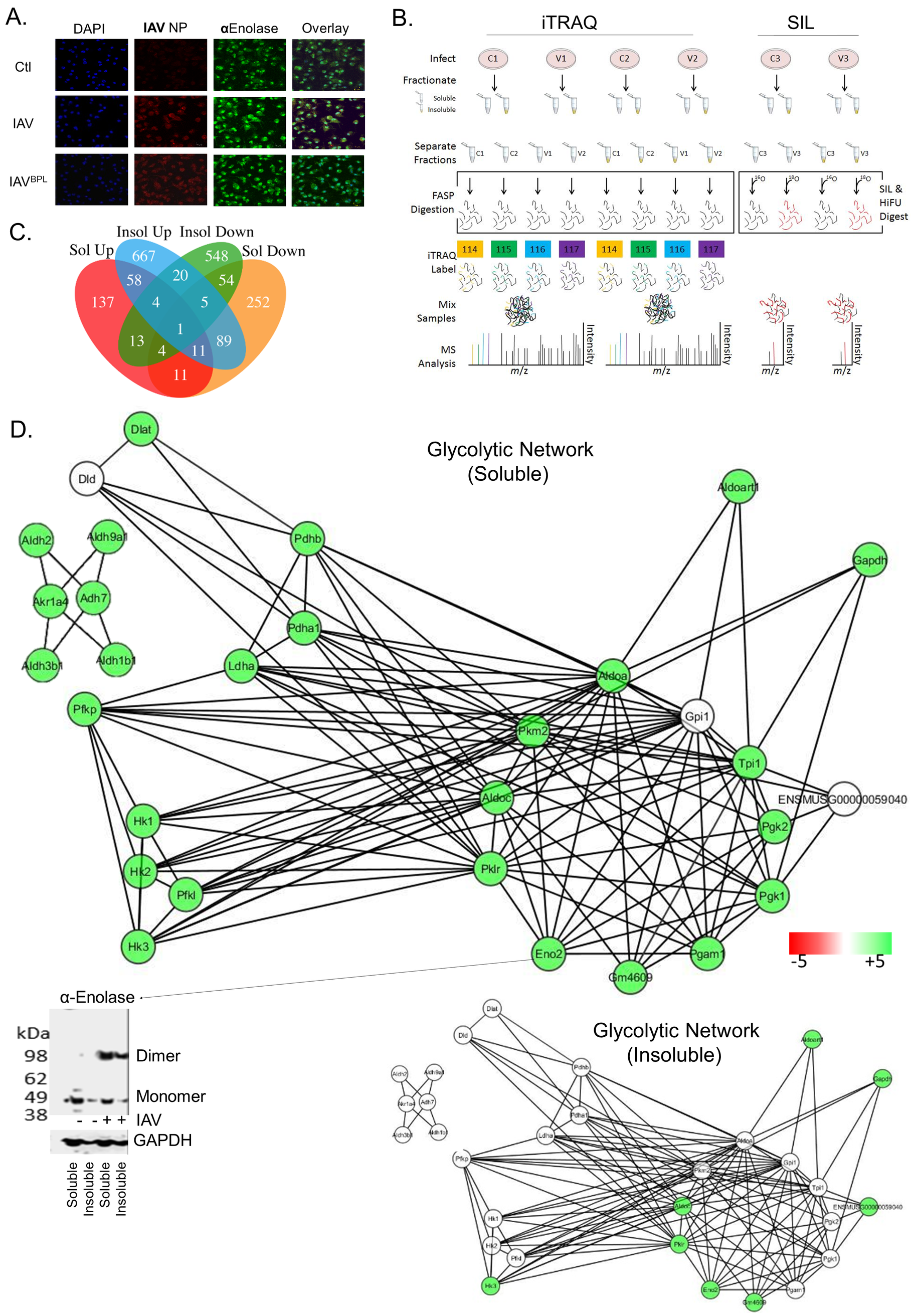

Supplement: S1 Fig — DC were left untreated (Ctl) or infected for 17 hours at MOI 5 with viable virus (IAV). (A) DC were also infected for 17 hours at MOI 5 with β-propiolactone inactivated virus (IAVBPL). DC were fixed and stained for DAPI, influenza nuclear protein or murine α-enolase protein and visualized with confocal microscopy. (B) Control uninfected cells or IAV-infected DC (MOI 5 pfu for 17 hours) were separated into soluble and insoluble fractions. The iTRAQ labeled samples were subjected to FASP digestion, while the SIL samples received trypsin-catalyzed 18O/16O labeling. The samples were desalted with C18 SPE, processed with a custom RPLC system and analyzed with a Velos Orbitrap mass spectrometer (iTRAQ) or LTQ-Orbitrap (SIL). (C) Venn-diagram depicting the distinct total number of proteins identified by iTRAQ and SIL, as well as the overlapping number of proteins. Venn-diagram illustrating the significant number of proteins identified by iTRAQ and SIL in soluble and insoluble fractions that were upregulated and downregulated as well as the overlapping number of proteins. (D) Both soluble and insoluble SIL DC proteomes were submitted to DAVID and PPI spider to define glycolytic protein-protein interaction networks. The glycolytic network was put into Cytoscape and integrated with quantitative data from the proteomic analysis. Alpha enolase increased in soluble and insoluble (inset) proteomes and was validated with immunoblotting revealing the monomer and dimer increased in both soluble (S) and insoluble (I) networks. (TIF) [file ppat.1008957.s001.tif]

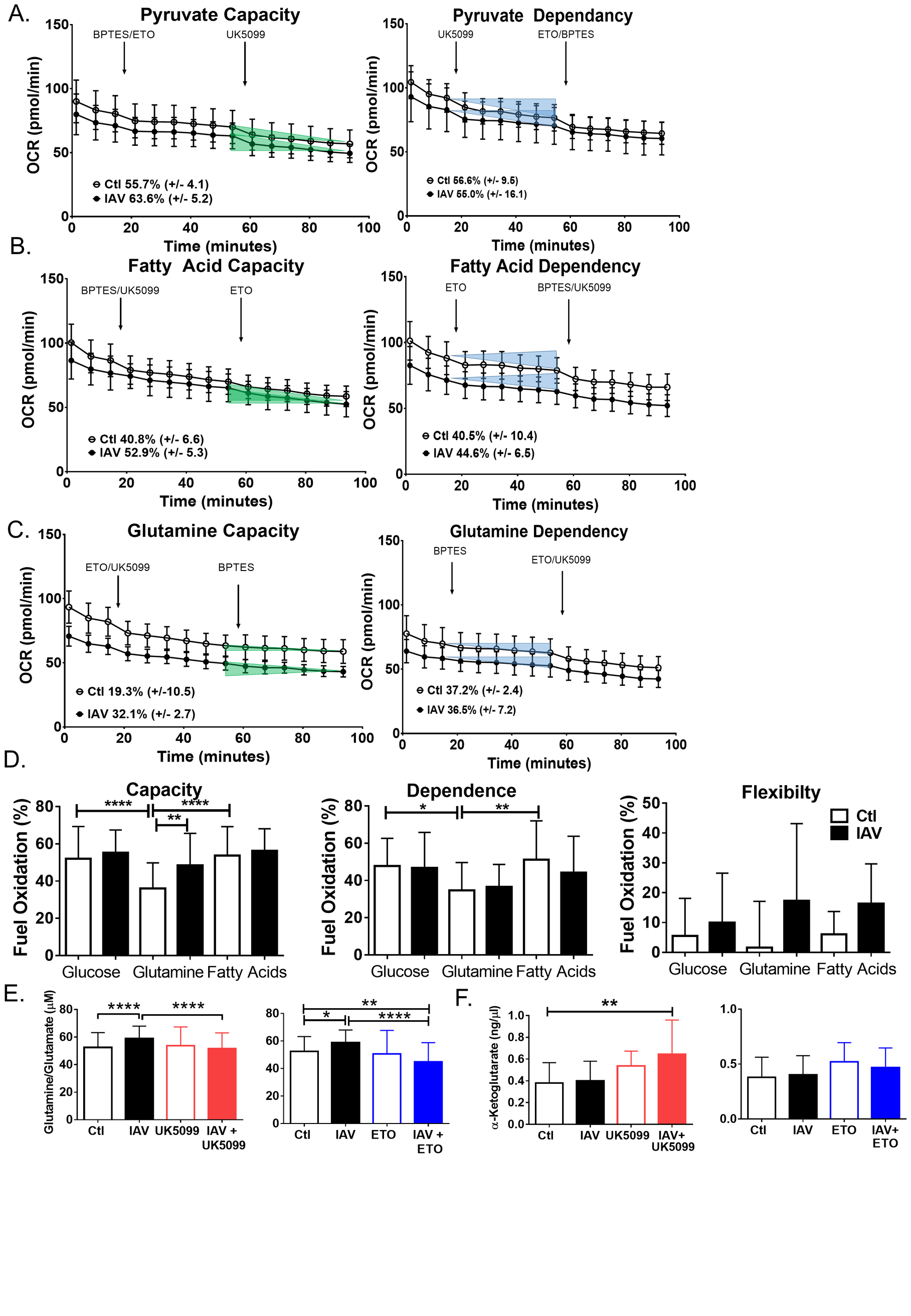

Supplement: S2 Fig — DC were left untreated (Ctl) or infected for 17 hours at MOI 5 with viable virus (IAV). (A-D) The rates of pyruvate, glutamine, or long chain fatty acids oxidation for respiration were calculated as the percentage of inhibition of oxygen consumption by UK5099, BPTES, or etomoxir, which are inhibitors of mitochondrial pyruvate carrier, glutaminase, and carnitine palmitoyltransferase 1A, respectively. Capacity for a specific substrate to drive respiratory OCR was tested by determining baseline OCR, inhibiting the 2 off target substrates determining OCR, and inhibiting import of the target metabolite. Percent capacity is one minus the baseline OCR less the off-target OCR divided by the baseline OCR less the OCR after all targets inhibited times 100. Dependency on a specific substrate was tested as above, reversing the inhibitor sequence, and the percent dependence was calculated by deducting the target OCR from the baseline and dividing by the baseline OCR less the OCR after all targets inhibited times 100. Fuel Flexibility was calculated as the difference between capacity and dependency. The average capacity of uninfected or infected DC to use either pyruvate, glutamine, or long chain fatty acids was determined. The average dependence of uninfected or infected DC on the oxidation of either pyruvate, glutamine, or long chain fatty acids was determined. The average flexibility of DC to use either pyruvate, glutamine, or long chain fatty acids was determined for uninfected or infected. (E-F) DC were pretreated with UK5099, etomoxir (ETO), or BPTES +/- IAV for 17 hours, rinsed and lysed for quantification of intracellular glutamine (E) or α-ketoglutarate activity (F). The bar graphs represent the values of 4–5 independent experiments and presented as experimental mean +/- SD p-value <0.05 (*), p-value < 0.01 (**), and p-value < 0.0001 (****). B-D show one representative experiment of 5 independent experiments with corresponding capacity, dependence, and flexibility values [file ppat.1008957.s002.tif]

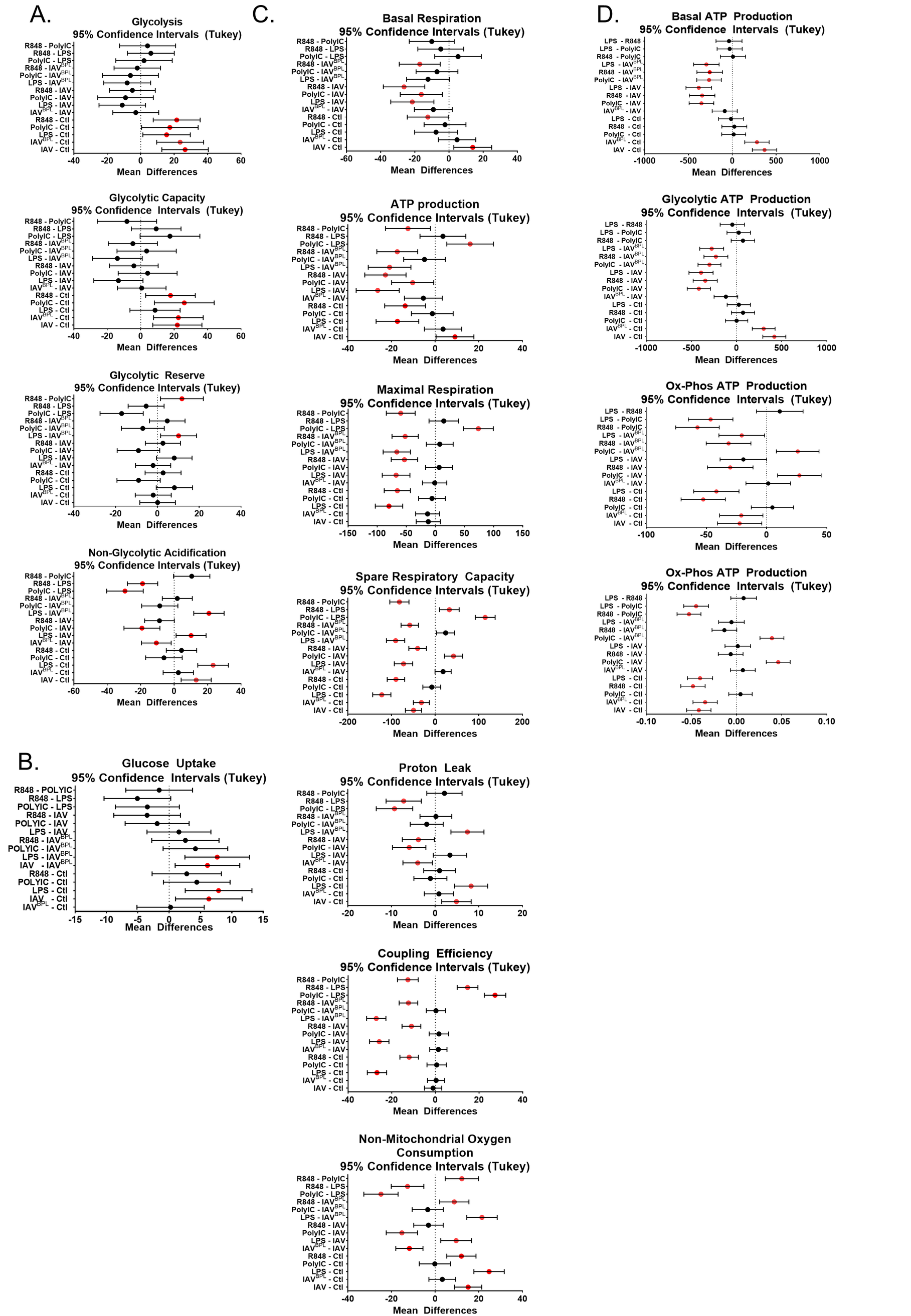

Supplement: S3 Fig — (A-D) DC were infected or treated with TLA agonists lipopolysaccharide (LPS), polyinosinic polycytidylic acid (PolyIC), or Resiquimod (R848) for 17 hours followed by metabolic analysis with a Seahorse Xfe96 Flux Analyzer. (A) Glycolytic function was tested while monitoring extracellular acidification rate (ECAR) with sequential injections of glucose, oligomycin (Oligo), and 2-Deoxy-D-glucose (2-DG) indicated by arrows. (B) Glucose uptake was monitored from the medium using a standard blood glucometer with glucose standard calibration curves. (C) Mitochondrial respiration was tested while monitoring oxygen consumption rates (OCRs) with sequential injections of oligomycin (Oligo), carbonyl cyanide-p-trifluoromethoxyphenylhydrazone (FCCP), and a mixture of rotenone and antimycin A (Rot/AntA) indicated by arrows. (D) DC maximal mitochondrial ATP changes induced by oligomycin plotted against maximal ATP changes upon glucose depletion determined by respirometry using Xfe96. The graphs represent the difference of the mean values from 3–4 independent experiments (3 ≥ technical replicates) and 95% confidence intervals. Significant differences among means were found with ANOVA followed by Tukey’s honest significant difference test, validated with Dunnett’s multiple comparison tests. Dashed line appears at 1, and red circles indicate confidence intervals do not overlap. (TIF) [file ppat.1008957.s003.tif]

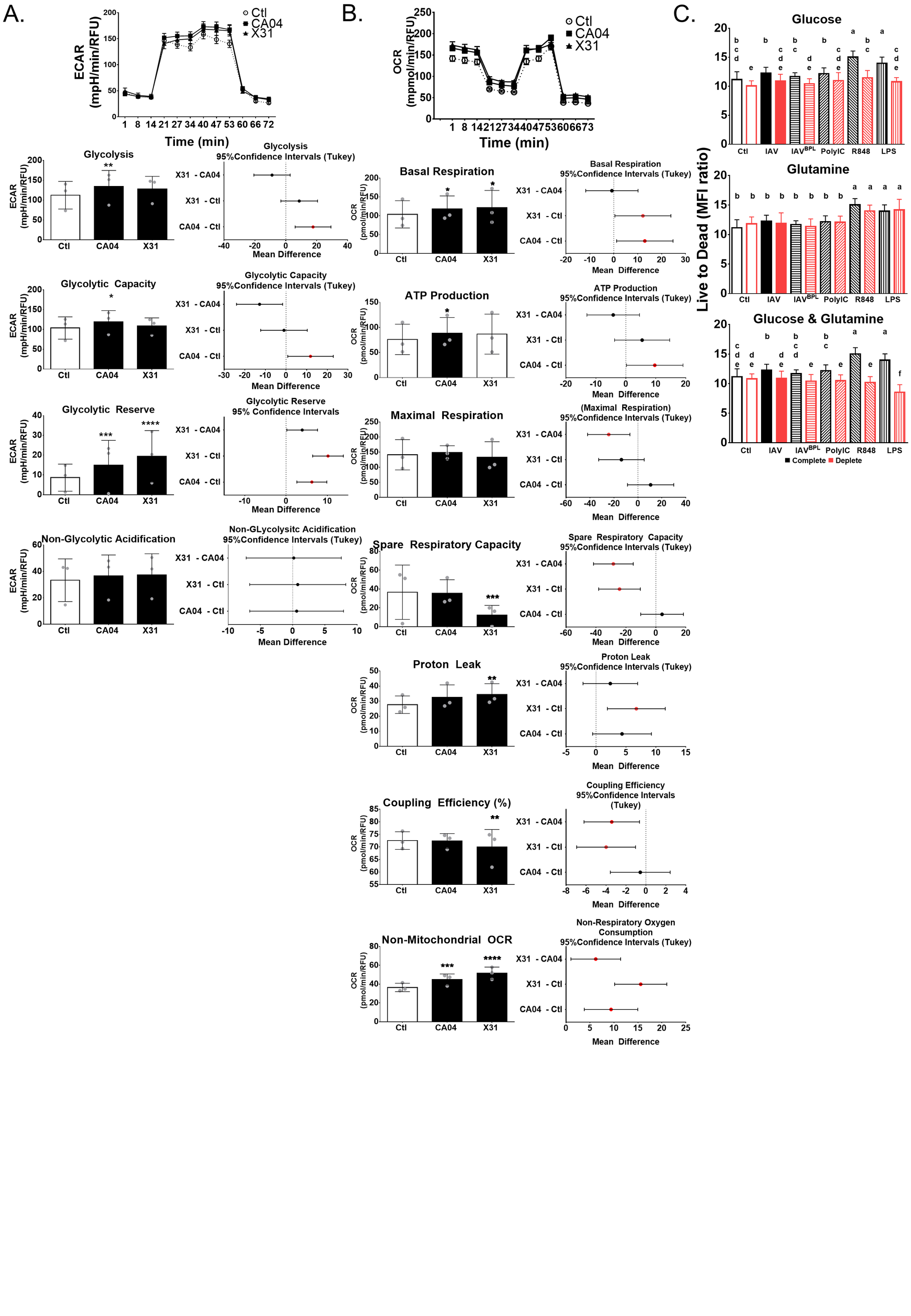

Supplement: S4 Fig — (A&B) DC were infected with CA04 or X31 influenza virus (MOI = 5) for 17 hours followed by metabolic analysis with a Seahorse Xfe96 Flux Analyzer. (A) Glycolytic function was tested while monitoring extracellular acidification rate (ECAR) with sequential injections of glucose, oligomycin (Oligo), and 2-Deoxy-D-glucose (2-DG) indicated by arrows. (B) Mitochondrial respiration was tested while monitoring oxygen consumption rates (OCR) with sequential injections of oligomycin (Oligo), carbonyl cyanide-p-trifluoromethoxyphenylhydrazone (FCCP), and a mixture of rotenone and antimycin A (Rot/AntA) indicated by arrows. The bar graphs represent mean and error bars standard deviation. Significant differences among means was found with ANOVA followed by Tukey's honest significant difference (Tukey's HSD) method. The difference of the means are plotted with Tukey's HSD 95% confidence intervals. The red circles indicate confidence intervals do not overlap. Tukey adjusted p values are symbolized by asterisks indicating adjusted p-values (* p≤0.05, ** p≤0.001, *** p≤0.0001, and **** p<0.0001). (C) DC growth medium was replaced with depleted medium 3 hours prior to infection or agonist treatment in blank or viral laden infection medium (MOI = 5) for 2 hours followed by a return to depleted medium for 17 hours. Viable, dead, and total DC were stained with Calcein-AM, ethidium homodimer and DAPI, respectively, and we measured fluorescence intensity using a microplate reader. Then mean fluorescence intensities (MFIs) and the ratio of live to dead DC were calculated. The graphs represent the mean values of 3 independent experiments +/- SD. Significant differences among means were found with ANOVA followed by Tukey's HSD, and results are summarized using compact letter display. Groups that are significantly different have different letters. (TIF) [file ppat.1008957.s004.tif]

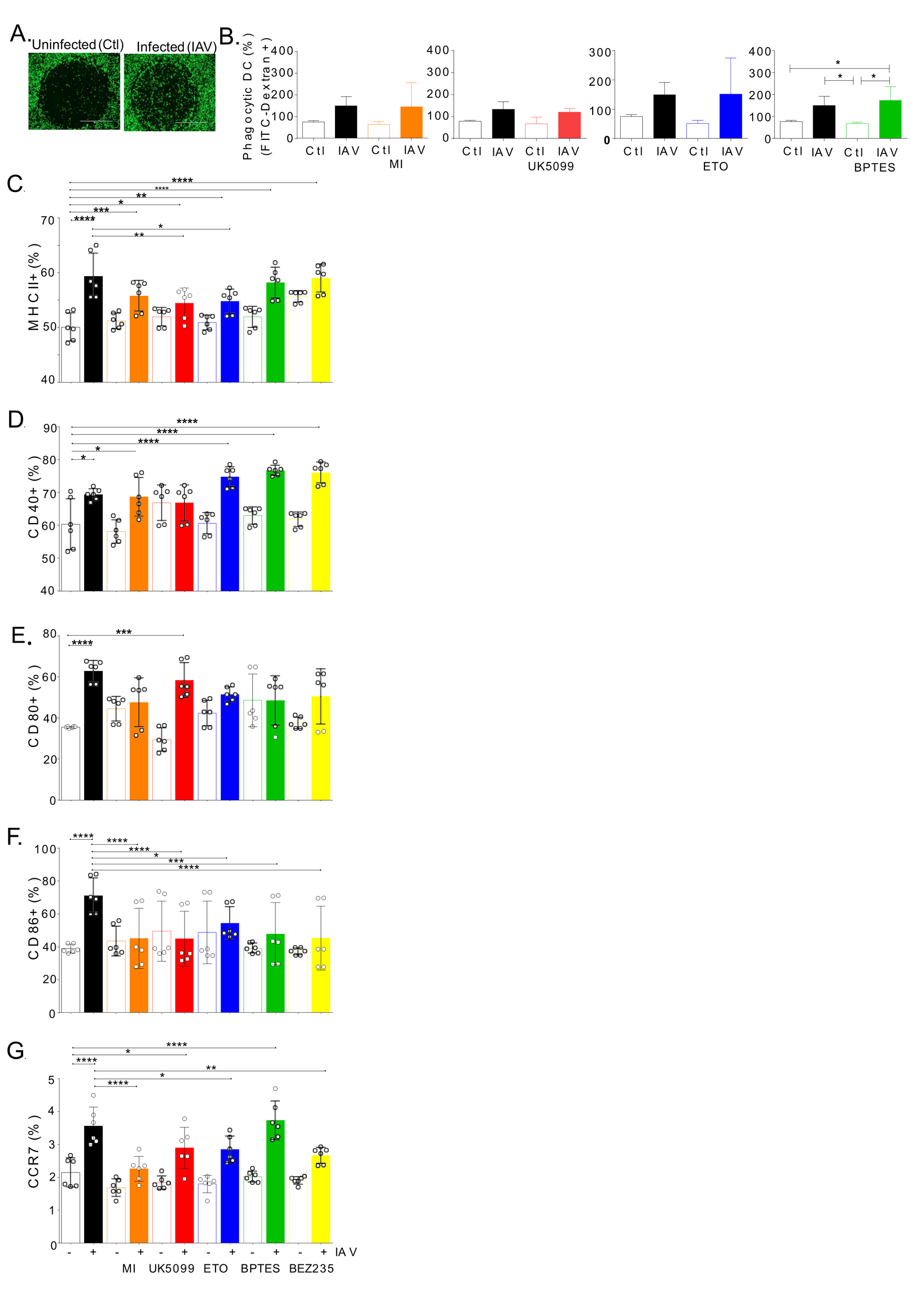

Supplement: S5 Fig — (A) DC were seeded on a precoated Oris 96-well motility plate allowed to adhere for 18+ hours followed by plug removal and infection (MOI = 5) for 17 hours. Live cells were stained with Calcein-AM, and images were acquired with EVOS. Two representative microscopy images of control and IAV motility are presented. (B-G) DC were pretreated with cMyc (2 μM), BPTES (3 μM), UK5099 (2 μM) or etomoxir (4 μM) and infected for 17 hours (MOI = 5) with IAV (solid bars) or left uninfected (open bars). (B) FITC-Dextran 40S (1mg/ml) was added and phagocytosis was terminated after 1 hour at 37°C. At 4°C, fluorescence per cell was measured using flow cytometry. (C-G) DC were stained for CD11c, CD40, CD80, CD86, MHCII surface markers and quantified by flow cytometry. We selected CD11c and MHCII positive and then gated on CD40, CD80 and CD86. (A-G) Graphs represent the mean values of 3 independent experiments with technical replicates (≥2). The error bars represent the SD. The statistical differences among means were found using ANOVA followed by Tukey's multiple comparisons test. Asterisks symbolize the adjusted p-values (* p≤0.05, ** p≤0.001, *** p≤0.0001, and **** p<0.0001). (TIF) [file ppat.1008957.s005.tif]

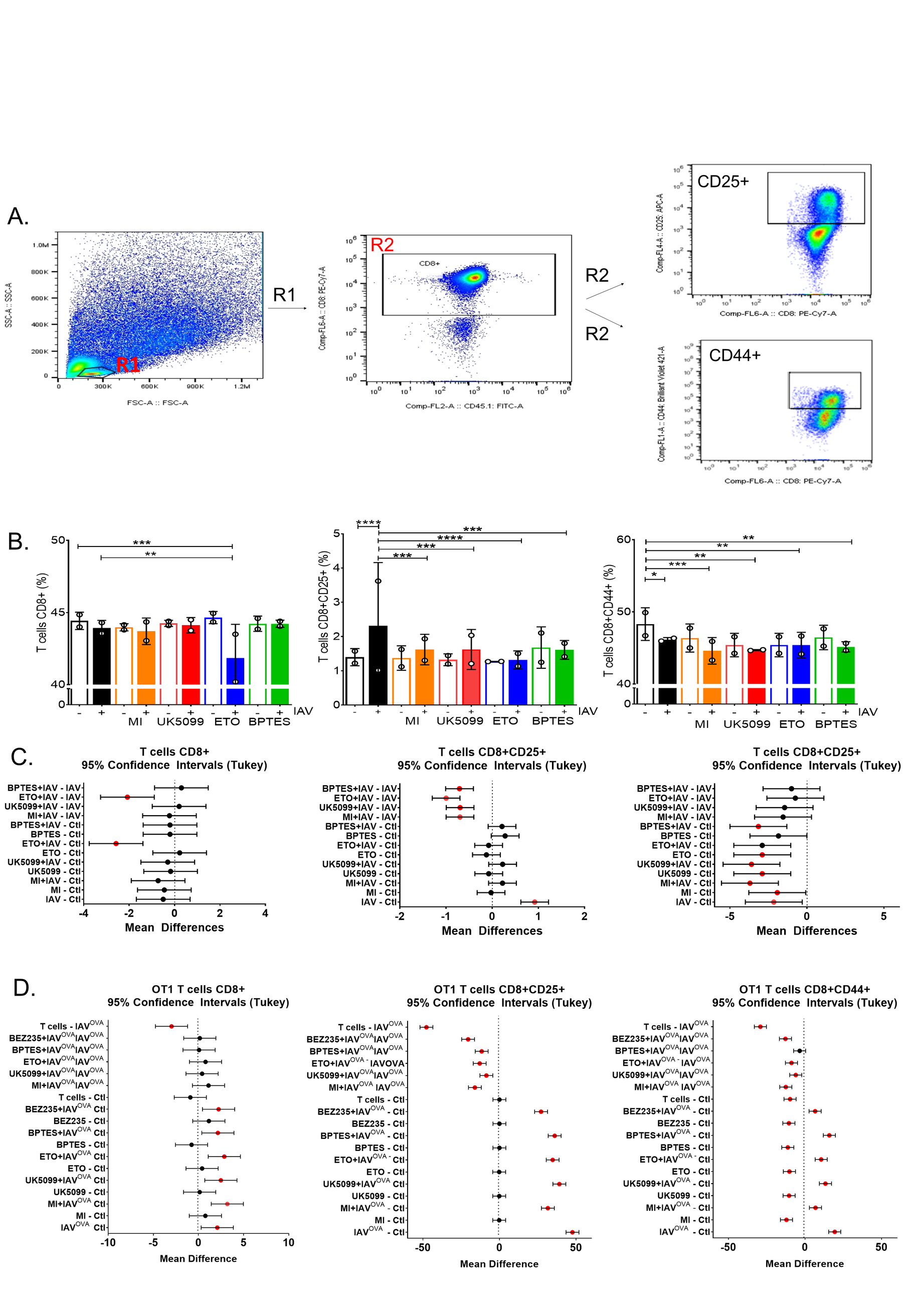

Supplement: S6 Fig — (C-F) CD8+ T cells were isolated by negative depletion from fresh splenocytes of homologously primed female C57BL/6 mice and co-cultured with DC at a 5:1 ratio of T cells to DC for 24 hours. Cells were fixed and stained for surface markers and quantified and compensated for using FlowJo software (2 additional independent experiments were performed on different instruments and quantified/compensated using different software. These experiments produced similar trends but were excluded due to differences in compensation methods). (C) Representative FACS plots with gating strategy. (D) Bar graphs represent the mean values of 2 independent experiments +/- SD. Statistical differences among means was found with ANOVA followed by Tukey's HSD with asterisks indicating adjusted p-values (* p≤0.05, ** p≤0.001, *** p≤0.0001, and **** p<0.0001). (E) Tukey's HSD results summarized with the circles representing the mean difference and the error bars the 95% confidence intervals. (F) Tukey's HSD results to from Fig 5D are summarized with the circles representing the mean difference and the error bars the corresponding 95% confidence intervals. (TIF) [file ppat.1008957.s006.tif]

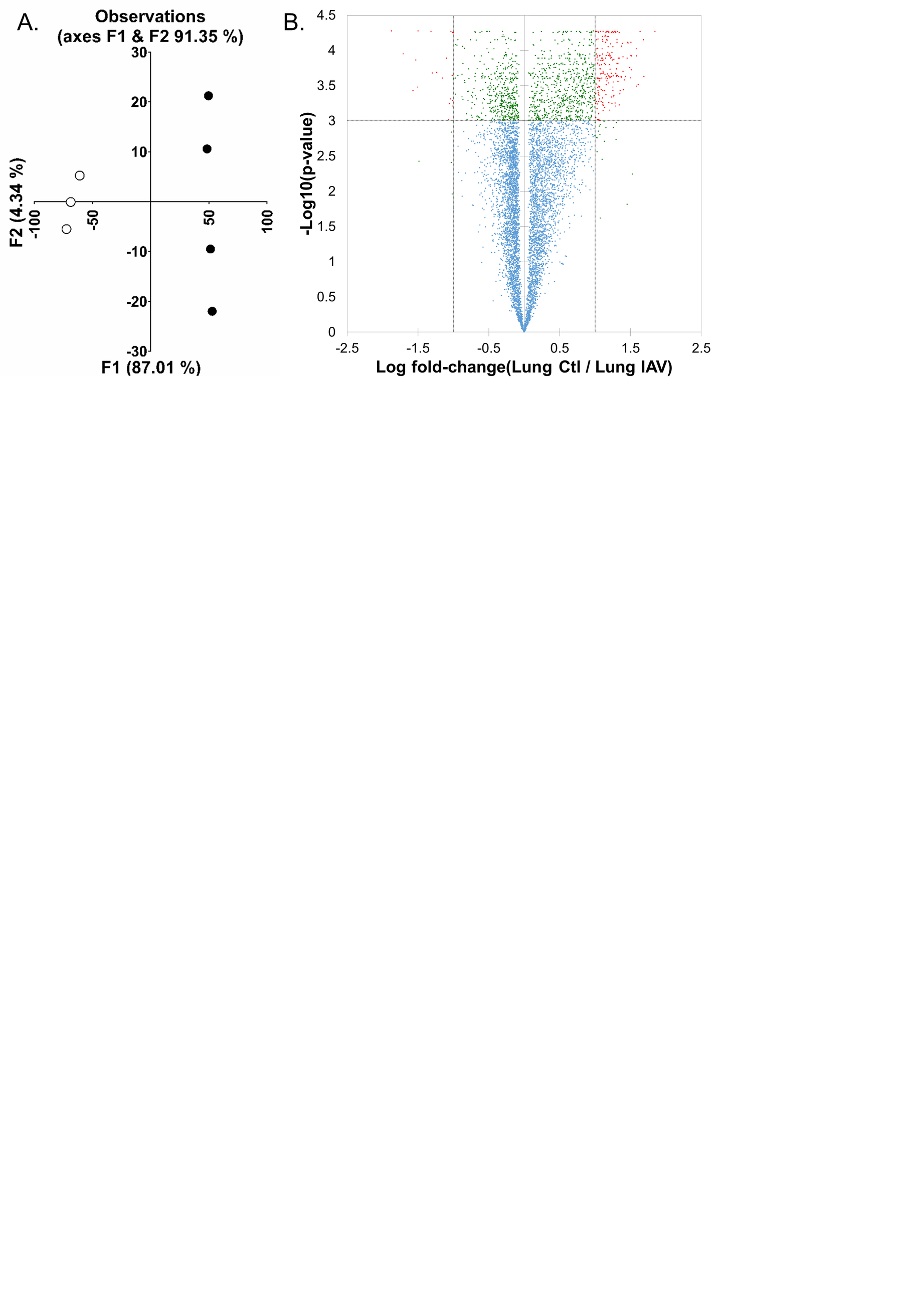

Supplement: S7 Fig — At zero or nine days following intranasal infection of mice with IAV at (EID50: 2000), the lungs were homogenized, and cells extracted. Cells were antibody stained for TipDC surface markers CD11b, Ly6c, GR-1, and MHCII. TipDC controls (Ctl) from day 0 were sorted based on high levels of CD11b, Ly6c, and GR-1. TipDC from day 9 of the IAV infection (IAV) were sorted based on high levels of CD11b, Ly6c, GR-1, and MHCII. cDNA libraries were generated from RNA, sequenced, and narrowed to confidently identified transcripts. (A) Unsupervised multivariate principal component analysis (PCA), resulting in F1 and F2 with a cumulative percent variability of 78.56%. Each circle represents a TipDC transcriptome, the open circles are control, and the solid black circles are IAV. (B) Differential expression was determined using Tukey’s HSD test for multiple comparisons with a Benjamini-Hochberg post hoc false discovery rate correction. A total of 6642 transcripts were removed with nonspecific filtering to remove transcripts that were not modulated by IAV (i.e., 50% standard deviation threshold). Log10 p-values are plotted against the Log2 ratio. (TIF) [file ppat.1008957.s007.tif]
