# Supplementary material for: Dynamic metabolic reprogramming in dendritic cells: An early response to influenza infection that is essential for effector function
Source: PLoS Pathog. 2020 Oct 26;16(10):e1008957. doi: 10.1371/journal.ppat.1008957 (PMC7707590; doi:10.1371/journal.ppat.1008957)
Supplement: S1 Table — The intensities of the isobaric tag reporter ions were quantified by using the MASIC tool with the exclusion of missing reporter-ion channels or by calculating the SIL ratio for each peptide pair after accounting for singly or doubly labeled species in the 16O/18O ratio and correcting for labeling efficiency. Then, the MS/MS data were searched and filtered by using 0.5% FDR; peptides passing the filter were quantified. Then, peptides-to-protein rollup was performed. (DOCX) [file ppat.1008957.s010.docx]

| Fraction | Up | No Change | Down | Total |
| --- | --- | --- | --- | --- |
| Insoluble | 855 | 2547 | 649 | 4051 |
| Soluble | 239 | 2003 | 427 | 2669 |
| Both | 74 | 1103 | 64 | 1241 |
