# Supplementary material for: Dynamic metabolic reprogramming in dendritic cells: An early response to influenza infection that is essential for effector function
Source: PLoS Pathog. 2020 Oct 26;16(10):e1008957. doi: 10.1371/journal.ppat.1008957 (PMC7707590; doi:10.1371/journal.ppat.1008957)
Supplement: S2 Table — Influenza virus strain A/PuertoRico/68/34 (IAV) was added to DC for 2 hours, infection medium was replaced, and the infection proceeded for 17 hours followed by cell lysis and protein extraction. Proteins were labeled with SIL or iTRAQ subjected to LC-MS/MS. Both proteomes were combined, redundancies removed, and confidently identified peptides with abundance changes of 2-fold or greater linked to protein identifiers. The lists of upregulated and downregulated proteins were submitted to the Database for Annotation, Visualization, and Integrated Discovery (DAVID) v6.7 and mapped to KEGG pathways. Significantly enriched major metabolic pathways are listed with proteins, soluble (Sol) or insoluble (Insol) fraction and fold change (FC) designated. (DOCX) [file ppat.1008957.s011.docx]

|  | **Protein** | **Fraction** | **FC** |  | **Protein** | **Fraction** | **FC** |  | **Protein** | **Fraction** | **FC** |
| --- | --- | --- | --- | --- | --- | --- | --- | --- | --- | --- | --- |
| **Glycolysis** | aldehyde reductase | Insol | 2 | **Oxidative Phosphorylation** | ATPase 16 kDa proteolipid subunit | Insol | 2 | **Biosynthesis of Amino Acids** | 3-phosphoglycerate dehydrogenase | Insol | 1 |
|  | aldehyde reductase | Sol | -2 |  | ATP synthase beta subunit | Sol | -0.5 |  | 3-phosphoglycerate dehydrogenase | Sol | -3 |
|  | aldolase | Insol | 2 |  | ATP synthase alpha subunit | Insol | -2 |  | aconitase | Insol | 2 |
|  | enolase | Insol | 5 |  | ATP synthase alpha subunit | Sol | -3 |  | aconitase | Sol | -1 |
|  | galactose mutarotase | Insol | 2 |  | ATP synthase subunit f | Insol | 5 |  | aldolase | Insol | 1.5 |
|  | galactose mutarotase | Sol | -1 |  | ATP synthase, subunit b | Insol | -3 |  | aldolase A | Sol | 2 |
|  | glucose phosphate isomerase | Insol | 2 |  | ATPase | Insol | 3 |  | aldolase C | Sol | 2 |
|  | glucose phosphate isomerase | Sol | -1 |  | ATPase | Sol | -2.5 |  | aminoacylase | Insol | 2 |
|  | hexokinase | Insol | 2 |  | ATPase subunit A1 | Sol | -3 |  | aminoacylase | Sol | -1 |
|  | hexokinase | Sol | 1 |  | ATPase subunit C | Insol | 2 |  | aminotransferase | Sol | 2 |
|  | phosphofructokinase | Insol | 2 |  | ATPase subunit C1 | Sol | 2 |  | arginase | Sol | 1 |
|  | phosphofructokinase | Sol | -2 |  | ATPase subunit D1 | Sol | -2 |  | arginase | Insol | 3 |
|  | phosphoglucomutase | Insol | 3 |  | ATPase subunit D2 | Sol | -3 |  | argininosuccinate lyase | Insol | 2 |
|  | phosphoglucomutase | Sol | -1 |  | ATPase subunit F | Insol | 2 |  | argininosuccinate lyase | Sol | -2 |
|  | phosphoglycerate kinase | Insol | 2 |  | ATPase subunit H | Sol | -2 |  | argininosuccinate synthetase | Sol | -2 |
|  | phosphoglycerate kinase | Sol | -1 |  | cytochrome c oxidase | Insol | 2 |  | argininosuccinate synthetase | Insol | -1 |
|  | phosphoglycerate mutase | Insol | 2 |  | NADH dehydrogenase A6 | Insol | 2 |  | branched chain aminotransferase | Insol | 1 |
|  | phosphoglycerate mutase | Sol | 1 |  | NADH dehydrogenase S8 | Insol | 2 |  | enolase | Insol | 5 |
|  | pyruvate kinase | Insol | 2 |  | NADH dehydrogenase S2 | Insol | 2 |  | glutamate oxaloacetate transaminase | Sol | -2 |
|  | pyruvate kinase | Sol | 1 |  | NADH dehydrogenase S3 | Insol | 2 |  | glutamate oxaloacetate transaminase | Insol | 2 |
|  | triosephosphate isomerase | Sol | 1 |  | NADH dehydrogenase A2 | Insol | 1 |  | glutamine synthetase | Insol | 1 |
|  | triosephosphate isomerase | Insol | 2 |  | NADH dehydrogenase 4 | Insol | 3 |  | glutamine synthetase | Sol | -2 |
|  |  |  |  |  | NADH dehydrogenase | Insol | 2 |  | isocitrate dehydrogenase | Insol | 1 |
| **TCA Cycle** | citrate synthase | Insol | -2 |  | NADH-ubiquinone oxidoreductase | Insol | 3 |  | isocitrate dehydrogenase | Sol | 1 |
|  | dihydrolipoamide dehydrogenase | Insol | -2 |  | pyrophosphatase | Insol | 2 |  | methionine adenosyltransferase | Insol | 3 |
|  | dihydrolipoamide dehydrogenase | Sol | -2 |  | pyrophosphatase | Sol | -1 |  | methionine adenosyltransferase | Sol | -1 |
|  | isocitrate dehydrogenase | Insol | -2 |  | ubiquinol-cytochrome c reductase | Insol | -2 |  | phosphofructokinase | Insol | 1.5 |
|  | isocitrate dehydrogenase | Sol | 2 |  |  |  |  |  | phosphofructokinase | Sol | 1 |
|  | isocitrate dehydrogenase | Insol | -2 |  | 2-deoxy-D-ribose 5-phosphate aldolase | Insol | 2 |  | phosphoglycerate kinase | Insol | 2 |
|  | malate dehydrogenase | Insol | -2 | **Pentose Phosphate Cycle** | 2-deoxy-D-ribose 5-phosphate aldolase | Sol | 1 |  | phosphoglycerate kinase | Sol | -1 |
|  | malate dehydrogenase | Sol | 1 |  | 6-phosphogluconolactonase | Insol | 2 |  | phosphoglycerate mutase | Insol | 2 |
|  | oxoglutarate dehydrogenase | Insol | -2 |  | 6-phosphogluconolactonase | Sol | -2 |  | phosphoglycerate mutase | Sol | 1 |
|  | pyruvate dehydrogenase | Insol | -2 |  | aldolase | Insol | 1.5 |  | pyruvate kinase | Insol | 2 |
|  | pyruvate dehydrogenase | Insol | -2 |  | aldolase | Sol | 2 |  | pyruvate kinase, muscle | Sol | -1 |
|  | succinate dehydrogenase | Insol | -3 |  | glucose phosphate isomerase | Insol | 2 |  | ribulose-5-phosphate-3-epimerase | Insol | 2 |
|  | succinate-CoA ligase | Insol | -2 |  | glucose phosphate isomerase | Sol | -1 |  | ribulose-5-phosphate-3-epimerase | Sol | 1 |
|  | succinate-CoAligase | Insol | -2 |  | glucose-6-phosphate dehydrogenase | Insol | 2 |  | serine hydroxymethyltransferase | Insol | -2 |
|  |  |  |  |  | glucose-6-phosphate dehydrogenase | Sol | 2 |  | serine hydroxymethyltransferase | Sol | -3 |
| **Fatty Acid Beta oxidation** | acetyl-CoAacyltransferase | Insol | -6 |  | GTPase | Sol | 1 |  | transaldolase | Insol | 2 |
|  | acetyl-CoAacyltransferase | Sol | -2 |  | phosphofructokinase | Insol | 1.5 |  | transaldolase | Sol | -1 |
|  | acyl-CoA synthetase | Insol | 1 |  | phosphofructokinase | Sol | 1 |  | transketolase | Insol | 2 |
|  | acyl-CoA synthetase | Sol | -2 |  | phosphoglucomutase | Insol | 3 |  | transketolase | Sol | -1 |
|  | acyl-CoAdehydrogenase | Insol | -2 |  | phosphoglucomutase | Sol | -1 |  | triosephosphate isomerase | Insol | 2 |
|  | acyl-CoAdehydrogenase | Sol | -1 |  | phosphogluconate dehydrogenase | Insol | 3 |  | triosephosphate isomerase | Sol | 1 |
|  | acyl-CoAoxidase | Insol | 1 |  | phosphogluconate dehydrogenase | Sol | -1 |  |  |  |  |
|  | acyl-CoAoxidase | Sol | -2.5 |  | ribulose-5-phosphate-3-epimerase | Insol | 2 |  |  |  |  |
|  | aldehyde dehydrogenase | Sol | -2 |  | ribulose-5-phosphate-3-epimerase | Sol | 1 |  |  |  |  |
|  | aldehyde dehydrogenase | Insol | -2 |  | transaldolase | Insol | 2 |  |  |  |  |
|  | dodecenoyl-Co A δ isomerase | Insol | -2 |  | transketolase | Insol | 2 |  |  |  |  |
|  | enoyl CoAhydratase | Insol | -2 |  |  |  |  |  |  |  |  |
|  | enoyl CoAhydratase | Sol | -1 |  |  |  |  |  |  |  |  |
|  | hydroxyacyl-Co A dehydrogenase | Insol | -2 |  |  |  |  |  |  |  |  |
