# Supplementary material for: Dynamic metabolic reprogramming in dendritic cells: An early response to influenza infection that is essential for effector function
Source: PLoS Pathog. 2020 Oct 26;16(10):e1008957. doi: 10.1371/journal.ppat.1008957 (PMC7707590; doi:10.1371/journal.ppat.1008957)
Supplement: S3 Table — The proteome was queried for components of the pyruvate dehydrogenase complex and subunits reported with corresponding functions, fold changes, names, and localization to soluble (Sol) or insoluble (Insol). A summary of identified metabolic proteins that relocalized from the soluble to insoluble fraction or vice versa is provided. Fold enrichment significance was generated from DAVID functional annotation clustering and the biological significance indicated with p-value derived from KEGG pathways. Fold changes from non-unique proteins identified in the same metabolic pathway were averaged. (DOCX) [file ppat.1008957.s012.docx]

| **Subunit Function** | **Fold Change** | | **Subunit Name** | **Fraction** |
| --- | --- | --- | --- | --- |
| E1 Regulatory (Activator) | -2 | Pyruvate dehydrogenase kinase 1 | | Insol |
| E1 Regulatory (Activator) | -2 | Pyruvate dehydrogenase kinase 3 | | Insol |
| E1 Catalytic | -1 | Pyruvate dehydrogenase beta | | Sol |
| E1 Catalytic | -1 | Pyruvate dehydrogenase alpha | | Sol |
| E1 Catalytic | -2 | Pyruvate dehydrogenase beta | | Insol |
| E1 Catalytic | -2 | Pyruvate dehydrogenase alpha | | Insol |
| E3 Non-catalytic | -2 | Pyruvate dehydrogenase component X | | Insol |
| E2 Catalytic | 1 | Dihydrolipoamide S-acetyltransferase | | Insol |
| E2 Catalytic | -2 | Dihydrolipoamide S-acetyltransferase | | Sol |
| E3 Catalytic | 1 | Dihydrolipoamide dehydrogenase | | Sol |
| E3 Catalytic | -2 | Dihydrolipoamide dehydrogenase | | Insol |
| E1 Regulatory (Inhibitor) | -2 | Pyruvate dehydrogenase phosphatase | | Insol |
